# Temperature Dependence of the Polar and Lewis Acid–Base Properties of Poly Methyl Methacrylate Adsorbed on Silica via Inverse Gas Chromatography

**DOI:** 10.3390/molecules29081688

**Published:** 2024-04-09

**Authors:** Tayssir Hamieh

**Affiliations:** 1 Faculty of Science and Engineering, Maastricht University, P.O. Box 616, 6200 MD Maastricht, The Netherlands; t.hamieh@maastrichtuniversity.nl; 2 Laboratory of Materials, Catalysis, Environment and Analytical Methods (MCEMA), Faculty of Sciences, Lebanese University, Beirut P.O. Box 6573/14, Lebanon

**Keywords:** London dispersion equation, recovery fraction, polar enthalpy and entropy, Lewis’s acid–base parameters, transition temperatures, intermolecular separation distance

## Abstract

The adsorption of polymers on solid surfaces is common in many industrial applications, such as coatings, paints, catalysis, colloids, and adhesion processes. The properties of absorbed polymers commonly vary with temperature. In this paper, inverse gas chromatography at infinite dilution was used to determine the physicochemical characterization of PMMA adsorbed on silica. A new method based on the London dispersion equation was applied with a new parameter associating the deformation polarizability with the harmonic mean of the ionization energies of the solvent. More accurate values of the dispersive and polar interaction energies of the various organic solvents adsorbed on PMMA in bulk phase and PMMA/silica at different recovery fractions were obtained, as well as the Lewis acid–base parameters and the transition temperatures of the different composites. It was found that the temperature and the recovery fraction have important effects on the various physicochemical and thermodynamic properties. The variations in all the interaction parameters showed the presence of three transition temperatures for the different PMMA composites adsorbed on silica with various coverage rates, with a shift in these temperatures for a recovery fraction of 31%. An important variation in the polar enthalpy and entropy of adsorption, the Lewis acid–base parameters and the intermolecular separation distance was highlighted as a function of the temperature and the recovery fraction of PMMA on silica.

## 1. Introduction

The determination of the physicochemical properties of polymers adsorbed on oxides plays an important role in various industrial applications and chemical processes, including specific composites such as plastics, paper, and rubber [1]. The surface modification of polymers via adsorption on metallic oxides is crucial in several industrial processes and products [1,2,3,4,5,6,7,8,9,10]. Indeed, in a solvent, the adsorption of polymers on solid surfaces is very useful for the dispersion or aggregation of concentrated suspensions or slurries. The adsorption process can contribute to the strength of the polymer segment contacts in the areas of adhesives, coatings, and polymer composites. For example, the physicochemical and mechanical properties of polymer composites are not only affected by the adhesion strength between the polymer and the reinforced filler but also by variations in the temperature.

The behavior of polymer composites strongly depends on the values of the transition temperatures and, more particularly, on their glass transitions. Polymers have different types of behavior with respect to the glass temperature (*T*_g_) depending on the temperature in the glass and liquid states. Most physical properties, including, for example, the enthalpy, rheological, and other surface properties, change with the temperature and time until reaching equilibrium if polymers are heated below *T*_g_ [8,9,10,11,12]. This is directly related to the physical aging or structure relaxation of polymers or devices made of glassy polymers [13,14]. 

The determination of the physicochemical properties of polymers adsorbed on oxides is required to prevent their behaviors in contact with other solids, liquids, or gasses. The composite polymer/metallic oxides are very often used for the coatings of paintings or industrial packing. Polymer composites or nanocomposites, such as acrylate polymers or poly methyl methacrylate (PMMA), adsorbed on oxides can be used in many applications, such as artificial muscles, urban furniture, aeronautics, and microelectronics due to their high mechanical properties and high capacitance density [8,15,16,17,18,19,20,21]. 

Many studies have been interested in the determination of the physicochemical properties [22,23,24,25,26,27,28,29,30,31,32,33,34,35,36,37,38,39,40,41,42,43,44,45,46] and the glass transition temperatures [47,48,49,50,51,52,53,54,55,56,57,58] of PMMA via inverse gas chromatography (IGC) at infinite dilution. This technique was applied to quantify the interactions between polymers, composites, or oxides and organic molecules under infinite dilution conditions [22,23,24,25,26,27,28,29,30,31,32,33,34,35,36,37,38,39,40,41,42,43,44,45,46,47,48,49,50,51,52,53,54,55,56,57,58]. The Lewis acid–base properties of insulating thermoplastic and thermosetting polymer materials [13,14,47,48,49,50,51,52,53,54,55,56,57,58] and the solubility parameters in appropriate solvents were also determined via the IGC technique. Papirer et al. [59] have studied the effect of surface acid–base characteristics of PMMA adsorbed on alumina, whereas Hamieh et al. [55,56,57,58,60] have highlighted the presence of three transition temperatures of PMMA in the bulk phase and when adsorbed on silica and alumina.

However, the effect of the temperature change on the polar interactions and the Lewis acid–base properties near the transition temperatures of PMMA adsorbed on silica has not yet been studied in the literature. 

In this paper, we proposed using the IGC technique at infinite dilution to study the impact of temperature variation on the London dispersive and polar free interaction energy and Lewis’s acid–base parameters of a PMMA/silica system for different recovery fractions of PMMA adsorbed on silica, applying our new methodology. Indeed, this recent method uses the London dispersion equation [44,45] and proposes a new thermodynamic parameter, $P_{SX}$, using the deformation polarizability $\alpha_{0X}$ of the probe and the ionization energies of the solid $\varepsilon_{S}$ and the solvent $\varepsilon_{X}$ via the following relation:

(1) $$P_{SX}=\frac{\varepsilon_{S}\;\varepsilon_{X}}{\left(\varepsilon_{S}+\varepsilon_{X}\right)}\alpha_{0X}.$$

By using the parameter $P_{SX}$, it was possible to obtain accurate values of the free interaction energy between the solid surfaces and the adsorbed organic molecules. The separation between the dispersive and polar interaction energies of PMMA adsorbed on silica at various recovery fractions allowed us to obtain the polar enthalpy and entropy of adsorption and the surface acid–base parameters of the PMMA/silica composites, such as the enthalpic and entropic Lewis’s acid–base constants of the solid substrates.

## 2. Experimental Results

### 2.1. Polar Surface Interactions between PMMA/Silica and Solvents

The values of deformation polarizability $\alpha_{0X}$ and the ionization energies of the various n-alkanes and polar molecules adsorbed on PMMA and silica were taken from the Handbook of Physics and Chemistry [61]. Tables S1–S3 (in Supplementary Materials) give the values of $\alpha_{0X}$ and the parameter $\frac{3N}{{2\left(4\pi \varepsilon_{0}\right)}^{2}}P_{S-X}$ of the different solvents. The values given in Tables S1–S3 allow us to determine the polar free surface energy ($-∆G_{a}^{p}\left(T\right)$) of the polar probes adsorbed on the different solid composites. Tables S4–S8 give the obtained ($-∆G_{a}^{p}\left(T\right)$) relative to PMMA adsorbed on silica by varying the recovery fraction.

The results in Table S4 show that the silica particles present higher polar interaction free energy with the base solvents and lower interaction energy with the acidic solvents. This led us to the conclusion that silica particle surfaces exhibit stronger acidity than the other solid materials (PMMA/silica) and a lower basic character. 

**Figure 1:**
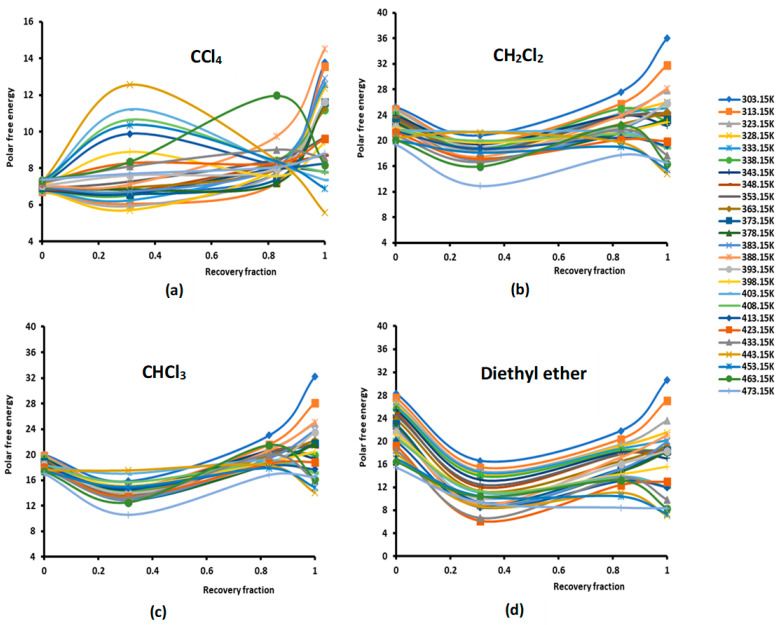

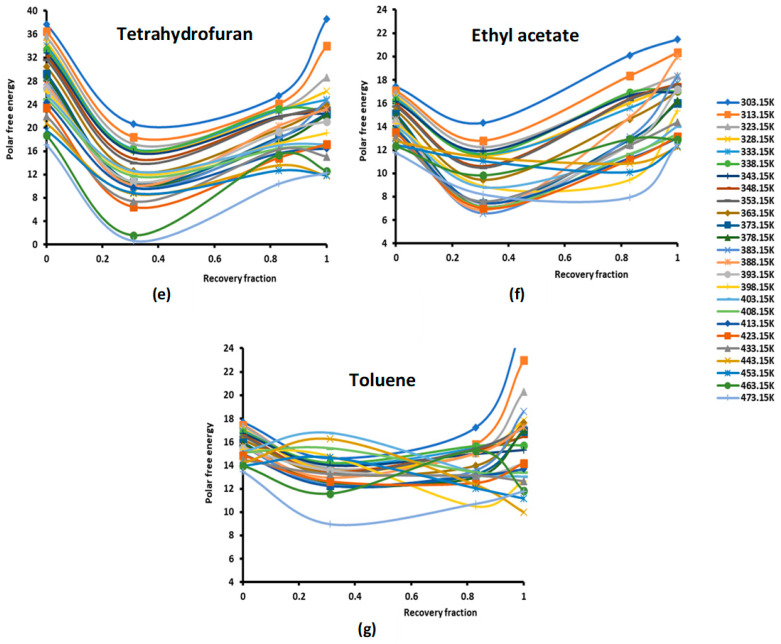
Evolution of the polar free interaction energy ($-∆G_{a}^{p}\left(T\right)$ (kJ/mol) of different polar solvents as a function of the recovery fraction of PMMA adsorbed on silica at different temperatures: (**a**) CCl4; (**b**) CH2Cl2; (**c**) CHCl3; (**d**) diethyl ether; (**e**) tetrahydrofuran (THF); (**f**) ethyl acetate; and (**g**) toluene.

On the contrary, the values in Table S5 relative to PMMA particles a show higher basic character and lower acid interaction energy. With a recovery fraction of 31% of PMMA adsorbed on silica, it was observed that the acidic character of silica decreases while its basicity increases to reach the maximum for a 100% recovery fraction (for one monolayer). The effects of the temperature and the recovery fraction of PMMA adsorbed on silica are shown in Figure 1 for the various polar solvents used in this study.

The study of the evolution of the polar free interaction energy of the various polar solvents adsorbed on the composites PMMA/silica with respect of the recovery fraction and temperature (Figure 1) showed a certain decrease in ($-∆G_{a}^{p}\left(T\right))$ until a recovery fraction of 31%, followed by an increase in the polar free interaction energy to reach a maximum when the coverage rate reaches 100% (in the case of a monolayer) at all temperatures, except for some specific temperatures higher than 380 K, corresponding to transition phenomena in PMMA, where a decrease in ($-∆G_{a}^{p}\left(T\right))$ against the coverage rate was observed. The same variations were found for the various adsorbed polar solvents.

The curves of ($-∆G_{a}^{p}\left(T\right)$) of the different solvents plotted in Figures S1–S7 clearly show the large effect of the temperature on the polar free energy with non-linear variations in the case of PMMA in bulk phase and when adsorbed on silica at different recovery fractions. This non-linearity of ($-∆G_{a}^{p}\left(T\right)$) is essentially observed near the transition temperatures of PMMA that are, respectively, highlighted at 333.15 K, 383.15, and 433.15 K, whereas the linearity is shown far from these transition temperatures. However, for all solvents, the linearity ($-∆G_{a}^{p}\left(T\right))$ is assured for silica particles, with an excellent linear regression coefficient very close to 1.000. It is also shown in Figures S1–S7 that the variations of ($-∆G_{a}^{p}\left(T\right))$ for the different coverage rates until the monolayer are, in general, limited between the two curves representative of PMMA in bulk phase and of silica particles, with the results of the monolayer approaching PMMA showing a mask effect exerted by PMMA on silica particles and limiting the role of silica in the interaction between the polar molecules and the solid surfaces.

**Figure 2:**
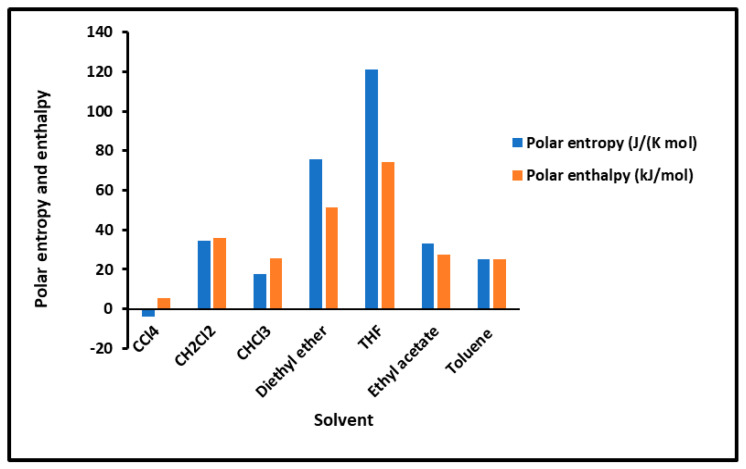
Values of the interaction enthalpy ($-∆H_{a}^{p}\left(T\right)(kJ/mol))$ and entropy ($-∆S_{a}^{p}\left(T\right)(JK^{-1}{mol}^{-1}))$ of polar solvents adsorbed on silica independent of the temperature.

**Figure 3:**
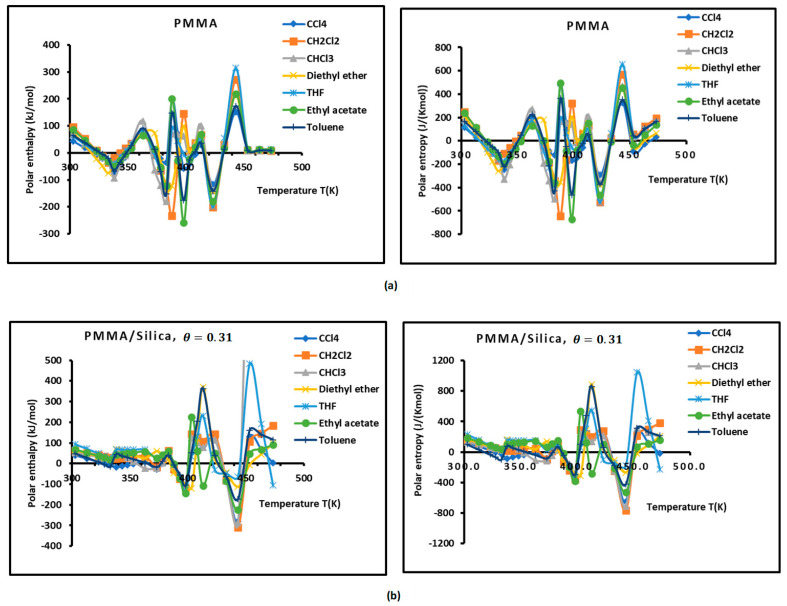

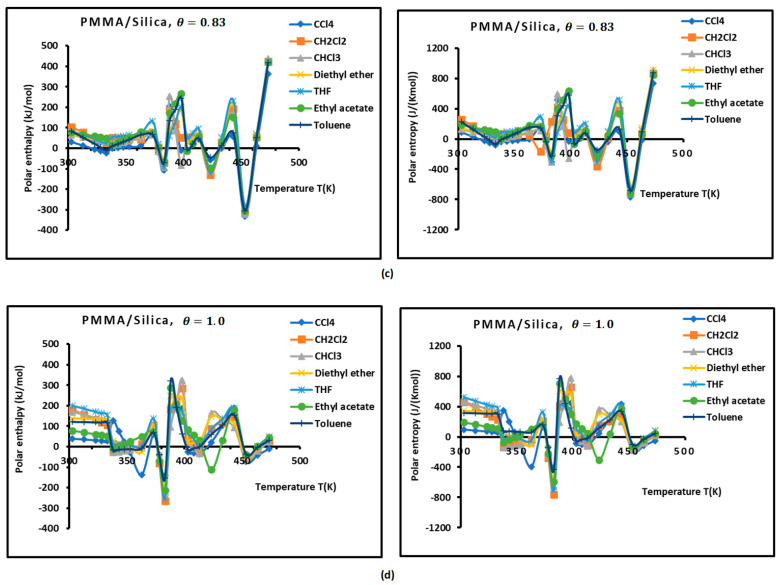
Variations of the interaction enthalpy ($-∆H_{a}^{p}\left(T\right))(kJ/mol)$ and entropy ($-∆S_{a}^{p}\left(T\right))(JK^{-1}{mol}^{-1})$ of polar solvents adsorbed on PMMA and PMMA/silica as a function of temperature: (**a**) PMMA; (**b**) PMMA/silica, θ=0.31; (**c**) PMMA/silica, θ=0.83; (**d**) PMMA/silica, θ=1.0.

To determine the polar enthalpy ($-∆H_{a}^{p}\left(T\right))$ and entropy ($-∆S_{a}^{p}\left(T)\right)$ of the interaction between solvents and composites, relations (8) and (9) were applied on the non-linear variations of ($-∆G_{a}^{p}\left(T\right))$ of the adsorbed polar solvents by taking small parabolic portions of the representative curves. The values of ($-∆H_{a}^{p}\left(T\right))$ and ($-∆S_{a}^{p}\left(T\right))$ are given in Tables S9–S13. An important difference in the behavior of the various solid surfaces is shown in Tables S4–S8, strongly depending on the temperature and on the coverage rate. To highlight the important effect of the temperature and the adsorption of PMMA on silica particles, the results are plotted in Figure 2 and Figure 3, giving the curves of the enthalpy ($-∆H_{a}^{p}\left(T\right))$ and entropy ($-∆S_{a}^{p}\left(T\right))$ of the polar solvents adsorbed on the various solid substrates as a function of the temperature by varying the coverage rate of the adsorption of PMMA on silica. The results in Figure 2 concerning the silica particles are plotted independently of the temperature; indeed, a perfect linearity is observed in the variations of the free polar energy ($-∆G_{a}^{p}\left(T\right)$ of all the adsorbed polar molecules, and constants values of ($-∆H_{a}^{p})$ and ($-∆S_{a}^{p}$) were obtained in the case of silica particles (Figure 2, Table S8).

However, the curves obtained with the various polymer PMMA composites in bulk and/or adsorbed phases plotted in Figure 3 show extreme non-linearity variations of ($-∆H_{a}^{p}\left(T\right))$ and entropy ($-∆S_{a}^{p}\left(T\right))$ versus temperature.

An intersting and original result is noted in the variations of polar enethalpy and entropy given in Figure 3, showing four minima of the various curves mentioning specific and particular temperatures. In previous papers [39,56,57,58,60], three transition temperatures were observed with PMMA and PMMA/silica at different coverages of PMMA on the silica particles, respectively, related to beta-relaxation ($T_{\beta }=60\;{}^{\circ}C$), glass transition ($T_{g}=110\;{}^{\circ}C$), and other liquid–liquid transitions ($T_{liq-liq}=160\;{}^{\circ}C$). A shift in these transition temperatures was observed in the case of the adsorption of PMMA on metallic oxides, reaching 20 °C when PMMA was adsorbed on silica. 

In this work, four temperatures in the case of PMMA were noted: $T_{\beta }=338.15\;K$; $T_{g}=383.15\;K$; $T_{3}=398.15\;K$; and $T_{liq-liq}=423.15\;K$ (Figure 3a, Table S9). The three previous transition temperatures were then confirmed by this study. However, the presence of a fourth temperature,$\;T_{3}=398.15\;K$, in the case of bulk PMMA deserves more thought.

In the case of PMMA/silica composites with a coverage rate θ=0.31, there are other shifted values of the transition temperatures. The new values are the following: $T_{\beta }=333.15\;K$; $T_{g}=373.15\;K$; $T_{3}=398.15\;K$; and $T_{liq-liq}=443.15\;K$ (Figure 3b, Table S10). It seems that this recovery fraction increases the liquid–liquid transition temperature by about 20 °C, with a decrease in the glass transition of about 10 °C relative to PMMA in bulk phase.

For a coverage fraction θ=0.83, the reported values are $T_{\beta }=333.15\;K$, $T_{g}=383.15\;K$, $T_{3}=403.15\;K$, and $T_{liq-liq}=423.15\;K$. The same values of PMMA in bulk phase were observed with a small variation of 5 °C for $T_{\beta }$ and $T_{3}$ (Figure 3c, Table S11).

However, for the case of one monolayer of PMMA on silica, identical values of the transition temperatures of bulk PMMA were noted, showing that the composite behavior of a monolayer is identical to that of PMMA in bulk phase (Figure 3d, Table S12). 

The presence of the different minima in Figure 3 is due to the reorganization and rearrangement of the various groups or chain segments of PMMA. The change in the retention mechanism of the probes and the adsorption variables at the transition temperatures are attributed to the increased molecular mobility of the polymer segments, allowing for the penetration of the probes into the polymer layer. Indeed, for polymers below the transition temperature, the penetration of the solute molecules in the bulk polymer is precluded, and retention proceeds only via surface adsorption. Then, at transition temperature, the penetration of the probe molecules begins, but due to an initially slow rate of diffusion of the solute into and out of the polymer, non-equilibrium conditions prevail. This can explain the decrease in the retention volume with temperature at the transition temperature. As temperature is increased, the diffusion coefficient rises sharply, which leads to equilibrium conditions. The polar and dispersive free energies of organic solvents, as well as the acid–base parameters, are submitted to the same variations obtained with the retention volumes of the solvents.

### 2.2. Enthalpic and Entropic Lewis’s Acid–Base Parameters 

The variations of the interaction enthalpy ($-∆H_{a}^{p}\left(T\right))$ and entropy ($-∆S_{a}^{p}\left(T\right))$ of the various polar molecules adsorbed on the different solid surfaces are given in Tables S4–S8 and plotted in Figure 2 and Figure 3. These results were used with relations 10 and 11 to determine the variations of the enthalpic acid–base parameters $K_{A}$ and $K_{D},$ as well as the entropic acid–base parameters $\omega_{A}$ and $\omega_{D}\;$ of PMMA and PMMA/silica at different recovery fractions as a function of the temperature. The calculated values of the acid–base parameter functions of the temperature for the different solid surfaces are given in Tables S6–S9. The obtained results show an important variation in the different acid–base parameters of PMMA and the composites PMMA/silica as a function of the temperature and coverage rate of the PMMA/silica system. The results in Table 1 show that PMMA exhibited higher basic surface (about 8 to 10 times more basic than acidic) and a lower acid character (about half of that found with the silica particles) (Table 2). However, the acid–base character of PMMA correlates strongly with the increase in the temperature, with sudden changes near the transition temperatures—i.e., negative values of the acid–base parameters around the transition temperature—certainly being due to the variation in the chemical group conformations in PMMA. Figures S8–S11 also show three to four secondary minima reflecting the presence of transition temperatures in PMMA and PMMA/silica systems. Figures S8–S15 clearly show the sudden variations of the acid–base parameters near the transition temperatures, and they also confirm these transition phenomena in the different composites of PMMA/silica. It can be observed that the acidity of PMMA/silica sightly varies when the recovery fraction increases from θ=0 to θ=0.83, whereas the basicity globally increases when the recovery fraction increases (Table 1 and Figures S8–S15). However, the results in Table S9 and Figure S11 give higher acid–base parameters in the case of the monolayer of PMMA on silica relative to silica particles and bulk PMMA. This special case of specific adsorption will probably create more surface chemical groups, increasing the number of acidic and basic groups in Lewis terms at the surface of PMMA/silica, which implies an increase in the Lewis acid–base parameters of the adsorbed PMMA on silica.

On the contrary, the case of silica gave acid–base parameters independent of the temperature (Table 2), showing a more acidic character than that of the other PMMA/silica surfaces.

### 2.3. London Dispersive Free Interaction Energies of PMMA/Silica

The new method applied in this paper, based on the London dispersive energy of interaction, allowed us to determine the London dispersive free energies of the adsorbed solvents on the PMMA/silica composites at different recovery fractions and temperatures by using the following London dispersion interactions.

(2) $$∆G_{a}^{d}\left(T\right)=A\left[\frac{3N}{{2\left(4\pi \varepsilon_{0}\right)}^{2}}P_{SX}\right]$$

**Figure 4:**
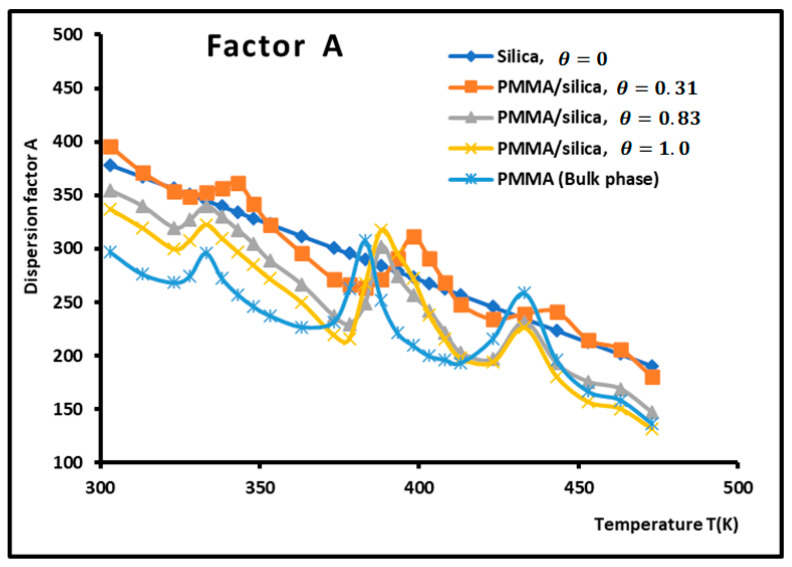
Variations of the dispersion factor A (SI unit) of PMMA/silica at different recovery fractions as a function of the temperature.

**Figure 5:**
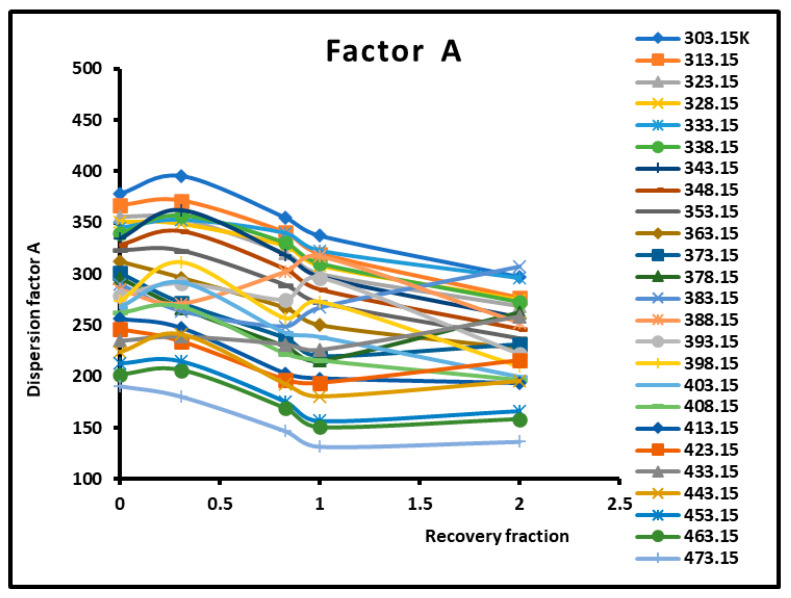
Variations of the dispersion factor A (SI unit) of different solid surfaces as a function of the recovery fraction for different temperatures.

The dispersion factor A was obtained by applying Equation (2) on the n-alkanes adsorbed on the various composite materials. The results are given in Table 3 and Figure 4 and Figure 5, which irrevocably show the presence of several transition temperatures located at the various maxima (in bold on Table 3) of the dispersion factor.

The values of the transition temperatures are identical to those previously obtained in this work and other studies. The adsorption of PMMA on silica is characterized by a transition temperature shift in the case of a recovery fraction θ=0.31. It is shown in Figure 5 that the limit of the dispersion factor was obtained for PMMA in bulk phase (and then for a recovery fraction θ≥2. 

**Figure 6:**
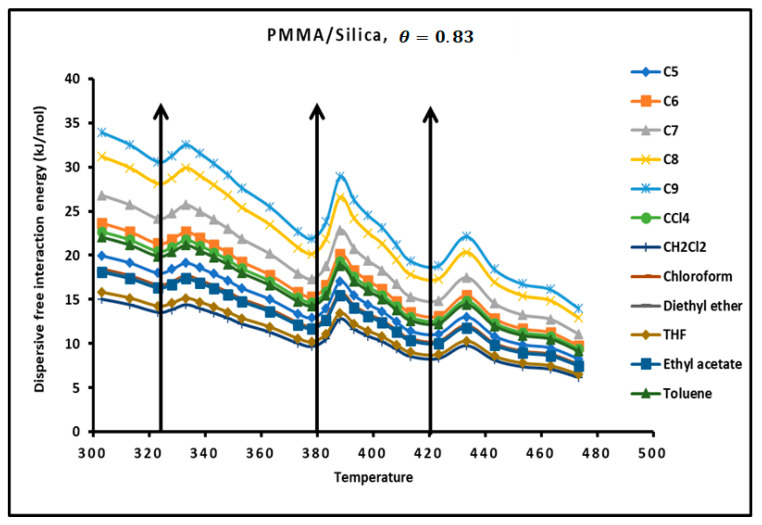
Evolution of the London free dispersive interaction energy ($-∆G_{a}^{d}\left(T\right))$ of organic solvents adsorbed on PMMA/silica for θ=0.83 as a function of the temperature.

The determination of the dispersion factor of the various PMMA/silica composites in Tables S9–S11 give the London dispersive energy of adsorption of the various organic solvents on PMMA (bulk phase) and PMMA/silica at different recovery fractions and temperatures. The transition temperatures are also highlighted in Tables S14–S17. Figure 6 gives an example of the variations of the London dispersive energy in the case of adsorption of PMMA on silica with θ=0.83. The same previous conclusions concerning the presence of the transition temperatures were observed for a recovery fraction of 31% of PMMA on silica.

### 2.4. Determination of the Separation Distance H between Solid Particles and Solvents

**Figure 7:**
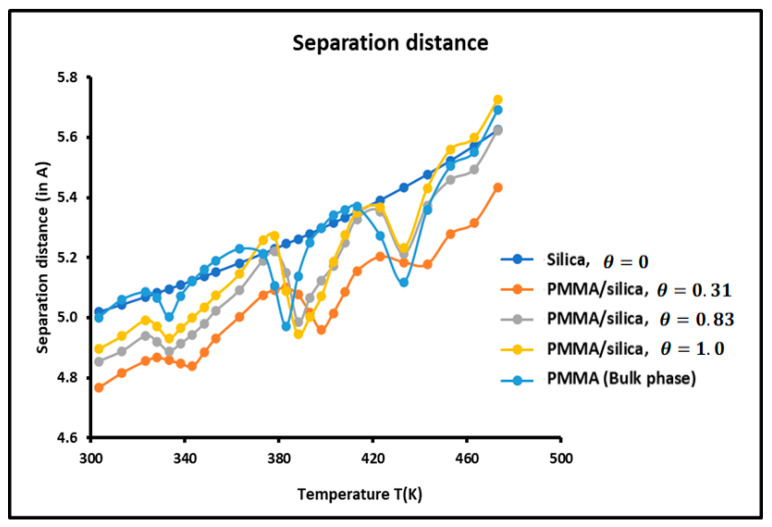
Variations of the separation distance *H* as a function of the temperature for the different solid surfaces.

The average separation distance H between the adsorbed solvents and the various solid surfaces was calculated using the experimental results and relations (5) and (6). The variations of H as a function of the temperature for the various solid substrates are plotted in Figure 7. 

It is observed that the variations of the separation H depend on the temperature, the nature of the solid surface, and the recovery fraction. H increased when the temperature increased. This conforms with the kinetic theory and the thermal agitation that has the effect of increasing the separation distance between particles. 

The larger separation H was found between silica particles and solvents, while the closest distance was obtained for a recovery fraction θ=0.31 of PMMA adsorbed on silica. When comparing the curves of Figure 6 and Figure 7, it is noted that for θ=0.31, the minimum of the dispersive energy corresponds to the maximum of the separation distance. Indeed, the minimum of attractive energy is necessary equivalent to the higher separation distance. Once again, the curves of Figure 7 show the presence of maxima at the transition temperatures.

## 3. Chromatographic Methods and Materials

The net retention time $t_{n}$ and volume $V_{n}$ of n-alkanes and polar molecules adsorbed on PMMA/silica surfaces were experimentally obtained by using inverse gas chromatography (IGC) at infinite dilution [22,23,24,25,26,27,28,29,30,31,32,33,34,35,36,37,38,39,40,41,42,43,44,45,46,47,48,49,50,51,52,53,54,55,56,57,58,59]. This led to the free energy of adsorption $∆G_{a}^{0}$ of adsorbed solvents on the solid surface (Equation (3)):

(3) $$∆G_{a}^{0}=-RT\;lnV_{n}+B\left(T\right),$$

where *T* is the absolute temperature of the chromatographic column containing the solid material, *R* the perfect gas constant, and B(T) is a constant depending on the temperature and reference characteristics referred to in the two-dimensional state of the adsorbed film.

$∆G_{a}^{0}$ is equal to the sum of the London dispersion component $∆G_{a}^{d}$ and the polar component $∆G_{a}^{p}$ of the free energy adsorption (Equation (4)):

(4) $$∆G_{a}^{0}=∆G_{a}^{d}+∆G_{a}^{p}.$$

Several chromatographic methods and models [29,30,32,33,34,35,36,37,38,39,42,43,44,45,46,47] have been used in the literature to determine the polar contribution $∆G_{a}^{p}$. The first separation method of the London dispersive and polar interactions between a solid surface and a polar molecule was proposed by Saint-Flour and Papirer [29,30], using the logarithm of the vapor pressure $P_{0}$ of the probes. Later, Schultz et al. used Fowkes relation [32] and the parameter of the dispersive component of the surface energy of the liquid solvent $\gamma_{l}^{d}$ to obtain the polar free energy of adsorption with the help of the surface area a of probes supposedly independent of the temperature. The notion of the deformation polarizability $\alpha_{0}$ was proposed by Donnet et al. [33], whereas the concept of the topological index $\chi_{T}$ was used by Brendlé and Papirer [34,35]. Hamieh criticized the different previous methods and models in several studies [38,39,42,43,44,45,46,47] by proving the thermal effect on the surface area of molecules and by showing the many difficulties and irregularities encountered when trying to obtain accurate values of the physicochemical parameters of the molecules adsorbed on the solid surfaces. This allowed us to propose a new method based on the London dispersive interaction energy between the solvents and the solid surfaces.

The London dispersion free energy can be expressed as

(5) $$∆G_{a}^{d}\left(T\right)=-\frac{\alpha_{0S}\;}{\;H^{6}}\left[\frac{3N}{{2\left(4\pi \varepsilon_{0}\right)}^{2}}\left(\frac{\varepsilon_{S}\;\varepsilon_{X}}{\left(\varepsilon_{S}+\varepsilon_{X}\right)}\alpha_{0X}\right)\right],$$

where N is the Avogadro’s number, $\varepsilon_{0}$ is the permittivity of vacuum, *S* denotes the solid particle, *X* is the solvent molecule separated by a distance H, and $\varepsilon_{S}$ and $\varepsilon_{X}$ are the respective ionization energies of the solid and the solvent $\varepsilon_{X}$. The new chromatographic chosen parameter of interaction between the solid and the solvent is given by

(6) $$P_{SX}=\frac{\varepsilon_{S}\;\varepsilon_{X}}{\left(\varepsilon_{S}+\varepsilon_{X}\right)}\alpha_{0X}.$$

In the case of n-alkanes $\left(C_{n}\right)$ adsorbed on the solid material, $RTlnVn\left(C_{n}\right)$ can be given by

(7) $$RTlnVn\left(C_{n}\right)=A\left[\frac{3N}{{2\left(4\pi \varepsilon_{0}\right)}^{2}}P_{SX}\left(C_{n}\right)\right]-B,$$

where A is given by

(8) $$A=\frac{\alpha_{0S}\;}{\;H^{6}}.$$

The straight line of n-alkanes representing the variations of $RTlnVn\left(C_{n}\right)$ against $\frac{3N}{{2\left(4\pi \varepsilon_{0}\right)}^{2}}P_{SX}\left(C_{n}\right)$ allowed us to determine the polar free energy $\left(-∆G_{a}^{p}(T)\right)$ of polar solvents adsorbed on PMMA/silica composites as a function of the temperature using the following equation:

(9) $$-∆G_{a}^{p}\left(T\right)=RTlnVn\left(X\right)-A\left[\frac{3N}{{2\left(4\pi \varepsilon_{0}\right)}^{2}}P_{S-X}\right]+B.$$

The polar enthalpy $-∆H_{a}^{p}(T)$ and entropy $-∆S_{a}^{p}(T)$ of organic molecules were then determined from the variations of $\left(-∆G_{a}^{p}(T)\right)$ using the following thermodynamic relations:

(10) $$∆H_{a}^{p}\left(T\right)=\frac{\partial \left(\frac{∆G_{a}^{p}\left(T\right)}{T}\right)}{\partial \left(\frac{1}{T}\right)},$$

(11) $$∆S_{a}^{p}\left(T\right)=-\left(\frac{\partial \left(∆G_{a}^{p}\left(T\right)\right)}{\partial T}\right).$$

The values of $-∆H_{a}^{p}(T)$ and $-∆S_{a}^{p}(T)$ of adsorbed polar solvents were obtained as a function of the temperature, and they allowed us to quantify the Lewis enthalpic acid–base constants $K_{A}(T)$ and $K_{D}(T)$ and the entropic acid–base parameters $\omega_{A}(T)$ and $\omega_{D}(T)$ with respect of the temperature:

(12) $$-∆H^{p}(T)=\;{DN\times K}_{A}(T)+AN\times K_{D}(T),$$

(13) $$-∆S^{p}\left(T\right)=\;{DN\times \omega }_{A}\left(T\right)+AN\times \omega_{D}\left(T\right),$$

where *AN* and *DN* are, respectively, the Gutmann electron donor and acceptor numbers of the polar solvents [62]. The used values were those corrected by Riddle and Fowkes [63].

The model organic molecules used as probes to quantify their interactions with the PMMA/silica composite were the following:

- The n-alkanes, such as n-pentane, n-hexane, n-heptane, n-octane, and n-nonane.
- The polar molecules, divided into three groups:
  ➢ Lewis’s acid solvents such as dichloromethane, chloroform, and carbon tetrachloride;
  ➢ Basic solvents such as ethyl acetate, diethyl ether, and tetrahydrofuran;
  ➢ Amphoterics such as toluene.

PMMA and silica solid particles with different recovery fractions of adsorbed polymer used in this work were the same solid materials previously characterized in other studies using other models and chromatographic methods [39,55,60]. The previous experimental data of RTlnVn of the various probes adsorbed on PMMA/silica composites obtained via the IGC technique at infinite dilution were used to study the effect of the temperature on the various thermodynamic and physicochemical parameters of the adsorption of PMMA on silica at different recovery fractions. A schematic diagram of the gas chromatograph is given by Figure S16.

The experimental conditions were optimized to obtain 20 mL/min of the flow rate of the carrier gas (helium). The temperatures of the injector and detector (Figure S16) were fixed at 200 °C. The infinite dilution of the probes was satisfied by using 1 µL Hamilton syringes and injecting very small quantities of the vapor probe, satisfying the limit of detection of the FID of high sensitivity, to practically realize the zero-surface coverage [46]. The columns containing the solid particles were preconditioned at 130 °C overnight to insure the total desorption of water molecules or any other residual impurities. The temperature of the column varied between 30 °C and 200 °C. Three injections of each solvent were carried out to determine the average value of the net retention time.

## 4. Conclusions

A new methodology was proposed to study the physicochemical properties of the composites constituted by the adsorption of PMMA on silica particles with various coverage rates between θ=0 (case of silica) and θ=1 corresponding to a monolayer. The inverse gas chromatography at infinite dilution was used by applying the London dispersion interaction energy to separate the polar and dispersive energy of the adsorbed solvents on the different solid surfaces. A new intrinsic thermodynamic parameter includes the deformation polarizability of solvents and the harmonic mean of the ionization energies of the solid surface and organic molecules. The determination of the polar interaction energy $∆G_{a}^{p}\left(T\right)$ as a function of the temperature relative to the various PMMA/silica composites led to the determination of the values of the polar enthalpy and entropy. It was shown that all polar surface variables depended on the temperature and the coverage rates of PMMA adsorbed on silica particles.

The Lewis enthalpic and entropic acid–base parameters were determined for the various solid surfaces. All acid–base parameters of PMMA and PMMA/silica were found to be strongly dependent on the temperature. Only those of silica particles did not depend on the temperature. Silica exhibited a higher acidic surface (twice as acidic as basic), whereas the acid–base parameters of PMMA in bulk phase showed a higher basic character, varying as a function of the temperature (eight times more basic than acidic, in general). The acidity of the PMMA/silica composites slightly varied versus the temperature relative to the highest values of the Lewis basic parameters. However, the basicity of PMMA/silica increased with the recovery fraction to reach the maximum for a monolayer of adsorption. It seemed that the acidity of the PMMA/silica for a monolayer reached the highest value.

The variations of the different thermodynamic and physicochemical parameters showed secondary minima and maxima, highlighting the presence of several transition temperature for PMMA in bulk phase and for the PMMA/silica systems with different recovery fractions, with a shift in such temperatures in the case of a recovery fraction equal to 31%.

This new study also determined the average separation distance between the organic solvents and the various solid surfaces. We showed that the separation distance comprises between 4 Å and 7 Å. We observed a slight variation in the intermolecular distance as a function of the temperature and the recovery fraction of PMMA on silica particles.

## Figures and Tables

**Table 1:** Values of the enthalpic acid–base parameters $K_{A}$, $K_{D}$, $K_{D}$*/*$K_{A}$, and $K_{D}+K_{A}$ and the entropic acid–base parameters $\omega_{A}$, $\omega_{D}$, $\omega_{D}\;$*/*${\;\omega }_{A}$, and ${(\omega }_{D}+\omega_{A})$ of PMMA and PMMA/silica at different recovery fractions as a function of the temperature.

**PMMA**								
**T(K)**	$K_{A}$	$K_{D}$	$K_{D}+K_{A}$	$K_{D}$ ** $/K_{A}$ **	** $10^{3}.\omega_{A}$ **	** $10^{3}.\omega_{D}$ **	${10^{3}(\omega }_{D}+\omega_{A})$	$\omega_{D}$ ** $/\omega_{A}$ **
303.15	0.478	3.881	4.358	8.120	0.90	18.20	19.10	20.15
313.15	0.317	2.523	2.840	7.953	0.56	7.52	8.08	13.40
323.15	0.090	0.664	0.754	7.413	−0.62	−3.15	−3.77	5.12
328.15	−0.122	−0.522	−0.644	4.293	−1.00	−4.91	−5.90	4.93
333.15	−0.153	−0.636	−0.789	4.160	−1.38	−6.06	−7.44	4.41
338.15	−0.144	−0.658	−0.802	4.568	−0.88	−7.59	−8.47	8.63
343.15	0.152	−0.695	−0.543	−4.565	−0.59	−8.31	−8.91	14.04
348.15	0.170	−0.529	−0.358	−3.103	−0.16	−1.87	−2.03	11.61
353.15	0.278	1.826	2.105	6.560	0.20	2.78	2.98	14.04
363.15	0.536	3.122	3.658	5.828	0.92	15.67	16.59	17.10
373.15	0.433	3.955	4.388	9.135	0.90	7.40	8.30	8.24
378.15	0.398	3.739	4.138	9.385	−1.99	−8.98	−10.97	4.52
383.15	0.186	1.527	1.713	8.217	−0.66	−6.52	−7.19	9.84
388.15	0.521	5.841	6.362	11.213	0.91	12.80	13.71	14.05
393.15	0.274	1.750	2.024	6.389	0.29	2.47	2.76	8.42
398.15	0.266	0.560	0.826	2.106	0.27	−1.89	−1.62	−6.93
403.15	0.220	−0.630	−0.410	−2.864	−0.45	−1.32	−1.77	2.92
408.15	0.174	0.800	0.975	4.595	0.22	−0.66	−0.44	−2.95
413.15	0.433	3.358	3.791	7.755	0.90	6.21	7.10	6.91
423.15	0.488	2.131	2.619	4.366	−4.56	−14.72	−19.27	3.23
433.15	0.543	0.904	1.447	1.665	0.66	−0.40	0.26	−0.61
443.15	1.755	7.262	9.016	4.138	5.88	19.88	25.76	3.38
453.15	0.111	1.044	1.155	9.419	0.60	−1.83	−1.22	−3.04
463.15	0.107	1.047	1.154	9.766	1.00	1.60	2.61	1.60
473.15	0.100	0.980	1.080	9.832	1.40	6.23	7.63	4.44
** PMMA/silica for θ=0.31 **								
**T(K)**	$K_{A}$	$K_{D}$	$K_{D}+K_{A}$	$K_{D}$ ** $/K_{A}$ **	** $10^{3}.\omega_{A}$ **	** $10^{3}.\omega_{D}$ **	${10^{3}(\omega }_{D}+\omega_{A})$	$\omega_{D}$ ** $/\omega_{A}$ **
303.15	0.884	3.819	4.704	4.318	2.11	10.71	12.82	5.06
313.15	0.704	2.319	3.023	3.291	1.53	5.84	7.37	3.81
323.15	0.519	0.770	1.288	1.484	0.95	0.97	1.91	1.02
328.15	0.424	−0.023	0.400	−0.055	0.65	−0.52	0.14	−0.79
333.15	0.327	−0.828	−0.501	−2.532	0.36	−0.73	−0.37	−2.02
338.15	0.789	0.508	1.298	0.644	1.97	−0.94	1.03	−0.48
343.15	0.781	0.387	1.168	0.495	1.95	−1.19	0.76	−0.61
348.15	0.773	0.264	1.037	0.341	1.92	−1.20	0.72	−0.63
353.15	0.764	0.139	0.903	0.181	1.90	−1.34	0.56	−0.71
363.15	0.747	−0.117	0.630	−0.157	1.85	−1.67	0.19	−0.90
373.15	2.013	2.013	4.026	1.000	4.85	15.21	20.05	3.14
378.15	0.425	0.594	1.020	1.397	0.84	−0.56	0.28	−0.67
383.15	0.781	0.387	1.168	0.495	1.95	−0.73	1.21	−0.38
388.15	−0.530	−1.038	−1.568	1.959	−0.57	−2.05	−2.62	3.61
393.15	−0.717	−1.493	−2.210	2.083	−1.54	−3.12	−4.66	2.03
398.15	−0.917	-2.588	−3.505	2.821	−2.39	−3.21	−5.60	1.34
403.15	0.584	7.647	8.231	13.084	−3.52	12.57	9.05	−3.58
408.15	1.006	11.211	12.216	11.149	2.39	18.37	20.76	7.67
413.15	2.013	14.818	16.831	7.362	4.85	21.18	26.03	4.37
423.15	−2.076	9.053	6.977	−4.361	−3.51	13.30	9.79	−3.79
433.15	−0.729	−2.522	−3.250	3.460	−1.26	−1.61	−2.87	1.27
443.15	0.550	−1.493	−0.944	−2.718	0.99	−1.72	−0.73	−1.74
453.15	4.266	9.254	13.520	2.169	10.38	−1.11	9.27	−0.11
463.15	1.213	15.550	16.763	12.821	3.76	10.71	14.47	2.84
473.15	−1.907	14.815	12.908	−7.768	−2.85	10.46	7.61	−3.67
** PMMA/silica for θ=0.83 **								
**T(K)**	$K_{A}$	$K_{D}$	$K_{D}+K_{A}$	$K_{D}$ ** $/K_{A}$ **	** $10^{3}.\omega_{A}$ **	** $10^{3}.\omega_{D}$ **	${10^{3}(\omega }_{D}+\omega_{A})$	$\omega_{D}$ ** $/\omega_{A}$ **
303.15	0.736	5.459	6.195	7.421	1.73	14.24	15.97	8.23
313.15	0.621	3.385	4.006	5.448	1.36	7.51	8.86	5.52
323.15	0.503	1.243	1.747	2.471	0.99	0.77	1.76	0.78
328.15	0.443	0.147	0.590	0.333	0.80	−1.40	−0.60	−1.74
333.15	0.381	−0.966	−0.584	−2.531	0.62	−1.92	−1.31	−3.12
338.15	0.579	−0.169	0.410	−0.292	1.07	−2.54	−1.47	−2.37
343.15	0.605	0.520	1.125	0.860	1.15	−1.89	−0.74	−1.64
348.15	0.632	1.220	1.851	1.931	1.23	−0.28	0.94	−0.23
353.15	0.659	1.929	2.588	2.929	1.30	1.74	3.04	1.33
363.15	0.714	3.379	4.093	4.732	1.46	5.79	7.24	3.97
373.15	0.893	3.854	4.747	4.316	1.91	0.69	2.60	0.36
378.15	0.315	−1.651	−1.336	−5.240	0.30	−0.70	−0.40	−2.34
383.15	0.605	0.520	1.125	0.860	1.15	2.31	3.46	2.01
388.15	0.778	22.143	22.921	28.465	−2.56	13.14	10.57	−5.12
393.15	0.962	14.877	15.839	15.464	1.76	12.13	13.89	6.90
398.15	2.200	7.518	9.718	3.417	4.89	11.61	16.50	2.38
403.15	0.595	−1.252	−0.657	−2.105	1.18	−2.16	−0.99	−1.84
408.15	0.743	1.285	2.028	1.730	1.54	0.41	1.95	0.27
413.15	0.893	3.854	4.747	4.316	1.91	6.66	8.57	3.50
423.15	−1.125	−1.531	−2.656	1.361	−1.46	−2.53	−3.99	1.74
433.15	0.602	0.223	0.824	0.370	1.38	−1.26	0.12	−0.91
443.15	2.369	6.543	8.912	2.762	5.41	10.87	16.28	2.01
453.15	−1.704	−4.480	−6.185	2.629	−2.26	−4.54	−6.80	2.01
463.15	0.679	2.897	3.577	4.264	1.26	2.74	4.00	2.18
473.15	4.310	23.213	27.523	5.386	2.06	2.73	4.80	1.33
** PMMA/silica for θ=0.1 **								
**T(K)**	$K_{A}$	$K_{D}$	$K_{D}+K_{A}$	$K_{D}$ ** $/K_{A}$ **	** $10^{3}.\omega_{A}$ **	** $10^{3}.\omega_{D}$ **	${10^{3}(\omega }_{D}+\omega_{A})$	$\omega_{D}$ ** $/\omega_{A}$ **
303.15	2.135	6.006	8.141	2.813	5.06	14.99	20.06	2.96
313.15	1.805	5.719	7.524	3.169	4.61	14.06	18.67	3.05
323.15	1.661	5.423	7.084	3.266	4.16	13.13	17.29	3.16
328.15	1.587	5.272	6.859	3.322	3.93	12.67	16.60	3.22
333.15	1.512	5.118	6.630	3.385	3.70	12.20	15.90	3.30
338.15	2.177	6.179	8.357	2.838	−2.30	15.92	13.62	−6.92
343.15	0.385	3.423	3.808	8.884	−3.19	12.37	9.19	−3.88
348.15	0.452	0.627	1.078	1.388	−2.59	4.01	1.42	−1.55
353.15	0.510	−1.015	−0.506	−1.992	0.51	4.36	4.87	8.54
363.15	0.611	−1.645	−-1.035	−2.695	0.75	13.22	13.96	17.68
373.15	0.385	−0.996	−0.611	−2.585	0.45	6.65	7.10	14.73
378.15	−0.439	−0.731	−1.169	1.666	−2.86	11.09	8.23	−3.88
383.15	−0.735	3.423	2.688	−4.659	−2.31	9.21	6.90	−3.99
388.15	1.235	21.076	22.312	17.060	2.59	19.86	22.46	7.66
393.15	1.188	18.884	20.072	15.892	2.47	21.30	23.78	8.61
398.15	1.141	13.057	14.197	11.448	2.35	18.65	21.01	7.93
403.15	0.887	−1.004	−0.117	−1.132	1.64	−1.93	−0.29	−1.17
408.15	0.558	−0.941	−0.383	−1.687	0.83	−1.86	−1.03	−2.25
413.15	0.224	−0.452	−0.228	−2.017	0.73	−1.81	−1.08	−2.49
423.15	0.786	2.985	3.771	3.798	1.57	4.81	6.38	3.08
433.15	1.060	9.697	10.756	9.149	2.21	13.41	15.62	6.08
443.15	1.340	13.578	14.918	10.131	2.85	15.73	18.58	5.53
453.15	−1.978	6.298	4.320	−3.184	−1.53	−1.92	−3.46	1.26
463.15	0.188	-0.611	−0.422	−3.242	0.23	−0.74	−0.51	−3.25
473.15	0.571	1.133	1.703	1.985	1.04	−0.74	0.31	−0.71

**Table 2:** Values of the enthalpic acid–base parameters $K_{A}$, $K_{D}$, $K_{D}$*/*$K_{A}$, and $K_{D}+K_{A}$ and the entropic acid–base parameters $\omega_{A}$, $\omega_{D}$, $\omega_{D}\;$*/*${\;\omega }_{A}$, and ${(\omega }_{D}+\omega_{A})$ of silica.

Solid Surface	$K_{A}$	$K_{D}$	$K_{D}+K_{A}$	$K_{D}$ $/K_{A}$	$10^{3}.\omega_{A}$	$10^{3}.\omega_{D}$	${10^{3}(\omega }_{D}+\omega_{A})$	$\omega_{D}$ $/\omega_{A}$
Silica	0.807	0.412	1.219	0.510	1.39	−1.32	0.07	−0.95

**Table 3:** Values of the dispersion factor A of the various PMMA/silica composites as a function of the temperature. The maxima of A are represented in bold.

T(K)	Silica, θ=0	PMMA/Silica, θ=0.31	PMMA/Silica, θ=0.83	PMMA/Silica, θ=1.0	PMMA
303.15	378.20	395.51	354.74	337.33	297.06
313.15	367.18	371.55	340.26	319.84	276.45
323.15	356.15	353.99	319.89	299.94	268.35
328.15	350.64	348.49	327.15	308.07	274.64
**333.15**	345.13	353.01	**340**	**322.86**	**296.16**
338.15	339.62	357.02	330.02	310.12	272.62
**343.15**	334.11	**361.76**	317.89	297.17	257.28
348.15	328.60	341.88	304.58	284.75	245.91
353.15	323.09	322.53	289.18	272.25	237.62
363.15	312.07	296.06	266.8	250.08	227
373.15	301.04	271.64	237.43	219.87	231.39
378.15	295.53	266.5	229.4	215.81	262.31
**383.15**	290.02	263.87	248.84	267.22	**307.3**
**388.15**	284.51	271.09	**302.26**	**317.52**	252.09
393.15	279.00	290.69	274.76	296.44	221.75
**398.15**	273.49	**311.45**	256.56	272.99	209.51
403.15	267.98	291.54	242.31	238.62	199.78
408.15	262.47	268.3	222.02	215.57	196.16
413.15	256.96	247.67	202.81	198.03	193.27
423.15	245.93	233.94	196.97	194.06	215.82
**433.15**	234.91	238.92	**231.59**	**226.19**	**258.37**
**443.15**	223.89	**241.02**	192.79	180.85	195.77
453.15	212.87	214.66	175.48	156.95	166.58
463.15	201.85	206.05	168.88	150.69	158.53
473.15	190.82	180.38	146.81	131.75	136.51

## Data Availability

There are no additional data.

## Supplementary Materials

The following supporting information can be downloaded at https://www.mdpi.com/article/10.3390/molecules29081688/s1: Table S1. Values of deformation polarizability and ionization energy of n-alkanes and polar molecules; Table S2. Values of the harmonic mean of the ionization energies $\frac{\varepsilon_{S}\varepsilon_{X}}{\left(\varepsilon_{S}+\varepsilon_{X}\right)}$ of silica and organic solvents and the parameter $\frac{3N}{{2\left(4\pi \varepsilon_{0}\right)}^{2}}P_{S-X}$ for the various organic molecules; Table S3. Values of the harmonic mean of the ionization energies $\frac{\varepsilon_{S}\varepsilon_{X}}{\left(\varepsilon_{S}+\varepsilon_{X}\right)}$ of PMMA and organic solvents and the parameter $\frac{3N}{{2\left(4\pi \varepsilon_{0}\right)}^{2}}P_{S-X}$ for the various organic molecules; Figure S1. Variations of the polar free interaction energy of CCl4 adsorbed on PMMA/silica a function of the temperature at different recovery fractions; Figure S2. Variations of the polar free interaction energy of CH2Cl2 adsorbed on PMMA/silica a function of the temperature at different recovery fractions; Table S4. Values of ($-∆G_{a}^{p}\left(T\right)$ kJ/mol) of polar molecules adsorbed on silica particles as a function of the temperature; Table S5. Values of ($-∆G_{a}^{p}\left(T\right)$ kJ/mol) of polar molecules adsorbed on PMMA particles as a function of the temperature; Table S6. Values of ($-∆G_{a}^{p}\left(T\right)$ kJ/mol) of polar molecules adsorbed on the system PMMA/silica as a function of the temperature for a recovery fraction θ=0.31; Table S7. Values of ($-∆G_{a}^{p}\left(T\right)$ kJ/mol) of polar molecules adsorbed on the system PMMA/silica as a function of the temperature for a recovery fraction θ=0.83; Table S8. Values of ($-∆G_{a}^{p}\left(T\right)$ kJ/mol) of polar molecules adsorbed on the system PMMA/silica as a function of the temperature for a recovery fraction θ=1; Figure S3. Variations of the polar free interaction energy of CHCl3 adsorbed on PMMA/silica as a function of the temperature at different recovery fractions; Figure S4. Variations of the polar free interaction energy of diethyl ether adsorbed on PMMA/silica as a function of the temperature, at different recovery fractions; Figure S5. Variations of the polar free interaction energy of THF adsorbed on PMMA/silica as a function of the temperature at different recovery fractions; Figure S6. Variations of the polar free interaction energy of ethyl acetate adsorbed on PMMA/silica as a function of the temperature at different recovery fractions; Figure S7. Variations of the polar free interaction energy of toluene adsorbed on PMMA/silica as a function of the temperature at different recovery fractions; Table S9. Values (in kJ/mol) of polar enthalpy ($-∆H_{a}^{p}\left(T\right)$ and ($-∆S_{a}^{p}\left(T\right)$ of polar solvents adsorbed on PMMA particles at different temperatures; Table S10. Values (in kJ/mol) of polar enthalpy ($-∆H_{a}^{p}\left(T\right)$ and ($-∆S_{a}^{p}\left(T\right)$ of polar solvents adsorbed on PMMA/silica particles at different temperatures for a recovery fraction of 31%; Table S11. Values (in kJ/mol) of polar enthalpy ($-∆H_{a}^{p}\left(T\right)$ and ($-∆S_{a}^{p}\left(T\right)$ of polar solvents adsorbed on PMMA/silica for θ=0.83 at different temperatures; Table S12. Values (in kJ/mol) of polar enthalpy ($-∆H_{a}^{p}\left(T\right)$ and ($-∆S_{a}^{p}\left(T\right)$ of polar solvents adsorbed on PMMA/silica for θ=1.0 (monolayer) at different temperatures; Table S13. Values (in kJ/mol) of polar enthalpy ($-∆H_{a}^{p}\left(T\right)$ and ($-∆S_{a}^{p}\left(T\right)$ of polar solvents adsorbed on silica particles at different temperatures; Figure S8. Evolutions of the enthalpic acid–base parameters $K_{A}$, $K_{D}$, $K_{D}$/$K_{A}$, and $K_{D}+K_{A}$ and the entropic acid–base parameters $\omega_{A}$, $\omega_{D}$, $\omega_{D}$/$\omega_{A}$, and ${(\omega }_{D}+\omega_{A})$ of PMMA as a function of the temperature; Figure S9. Evolutions of the enthalpic acid–base parameters $K_{A}$, $K_{D}$, $K_{D}$/$K_{A}$, and $K_{D}+K_{A}$, and the entropic acid–base parameters $\omega_{A}$, $\omega_{D}$, $\omega_{D}$/$\omega_{A}$, and ${(\omega }_{D}+\omega_{A})$ of PMMA/silica for θ=0.31 as a function of the temperature; Figure S10. Evolutions of the enthalpic acid–base parameters $K_{A}$, $K_{D}$, $K_{D}$/$K_{A}$, and $K_{D}+K_{A}$ and the entropic acid–base parameters $\omega_{A}$, $\omega_{D}$, $\omega_{D}$/$\omega_{A}$, and ${(\omega }_{D}+\omega_{A})$ of PMMA/silica for θ=0.83 as a function of the temperature; Figure S11. Evolutions of the enthalpic acid–base parameters $K_{A}$, $K_{D}$, $K_{D}$/$K_{A}$, and $K_{D}+K_{A}$ and the entropic acid–base parameters $\omega_{A}$, $\omega_{D}$, $\omega_{D}$/$\omega_{A}$, and ${(\omega }_{D}+\omega_{A})$ of PMMA/silica for θ=1.0 as a function of the temperature; Figure S12. Evolutions of the enthalpic Lewis acid parameter $K_{A}$ as a function of the recovery fraction and temperature; Figure S13. Evolutions of the enthalpic Lewis basic parameter $K_{D}$ as a function of the recovery fraction and temperature; Figure S14. Evolutions of the entropic Lewis acidic parameter $\omega_{A}$ as a function of the recovery fraction and temperature; Figure S15. Evolutions of the entropic Lewis basic parameter $\omega_{D}$ as a function of the recovery fraction and temperature; Table S14. Values (in kJ/mol) of the London free dispersive interaction energy ($-∆G_{a}^{d}\left(T\right)$ of organic solvents adsorbed on PMMA at different temperatures; Table S15. Values (in kJ/mol) of the London free dispersive interaction energy ($-∆G_{a}^{d}\left(T\right)$ of organic solvents adsorbed on PMMA/silica for θ=0.31 at different temperatures; Table S16. Values (in kJ/mol) of the London free dispersive interaction energy ($-∆G_{a}^{d}\left(T\right)$ of organic solvents adsorbed on PMMA/silica for θ=0.83 at different temperatures; Table S17. Values (in kJ/mol) of the London free dispersive interaction energy ($-∆G_{a}^{d}\left(T\right)$ of organic solvents adsorbed on PMMA/silica for θ=1.0 (monolayer) at different temperatures; Figure S16. Elements of a gas chromatograph.
