# Supplementary material for: Temperature Dependence of the Polar and Lewis Acid–Base Properties of Poly Methyl Methacrylate Adsorbed on Silica via Inverse Gas Chromatography
Source: Molecules. 2024 Apr 9;29(8):1688. doi: 10.3390/molecules29081688 (PMC11052169; doi:10.3390/molecules29081688)
Supplement: Supplementary file 1 [file molecules-29-01688-s001.zip › molecules-2949513-supplementary.pdf]

Supplementary Materials

Temperature Dependence of the Polar and Lewis Acid–Base Properties of Poly Methyl Methacrylate Adsorbed on Silica via Inverse Gas Chromatography

Tayssir Hamieh[1,2]

[1]Faculty of Science and Engineering, Maastricht University, P.O. Box 616, 6200 MD Maastricht, Netherlands
[2]Laboratory of Materials, Catalysis, Environment and Analytical Methods (MCEMA), Faculty of Sciences, Lebanese University, Hadath, Lebanon

Correspondence: Faculty of Science and Engineering, Maastricht University, P.O. Box 616, 6200 MD Maastricht, The Netherlands, E-mail: t.hamieh@maastrichtuniversity.nl

Table S1. Values of deformation polarizability and ionization energy of n-alkanes and polar molecules.

**Molecule**	ε_X **(eV)**	α_0 **(10^{-30} m^3)**	α_0 **(10^{-40} C m^2/V)**
n-pentane	10.28	9.99	11.12
n-hexane	10.13	11.90	13.24
n-heptane	9.93	13.61	15.14
n-octane	9.80	15.90	17.69
n-octane	9.71	17.36	19.32
CCl_4	11.47	10.85	12.07
$CHCl_3$	11.32	7.21	8.02
CH_2Cl_2	11.37	8.87	9.86
Diethyl ether	9.51	9.47	10.54
Tetrahydrofuran	9.38	8.22	9.15
Ethyl acetate	10.01	9.16	10.19
Toluene	8.83	11.80	13.13

Table S2. Values of the harmonic mean of the ionization energies $\frac{\varepsilon_S\,\varepsilon_X}{(\varepsilon_S+\varepsilon_X)}$ of silica and organic solvents and the parameter $\frac{3\mathcal{N}}{2(4\pi\varepsilon_0)^2}\mathcal{P}_{S-X}$ for the various organic molecules.

**Molecule**	$\frac{\varepsilon_S\,\varepsilon_X}{(\varepsilon_S+\varepsilon_X)}$	$\frac{3\mathcal{N}}{2(4\pi\varepsilon_0)^2}\mathcal{P}_{S-X}$

	$\frac{\varepsilon_S \varepsilon_X}{(\varepsilon_S + \varepsilon_X)}$ (10^{-19}J)	$\frac{3\mathcal{N}}{2(4\pi\varepsilon_0)^2}\mathcal{P}_{S-X}$ (10^{-15} SI)
n-pentane	7.274	58.992
n-hexane	7.226	69.814
n-heptane	7.162	79.135
n-octane	7.119	91.901
n-octane	7.089	99.919
CCl_4	7.623	67.151
$CHCl_3$	7.582	44.379
CH_2Cl_2	7.596	54.666
Diethyl ether	7.022	53.988
Tetrahydrofuran	6.977	46.564
Ethyl acetate	7.188	53.453

Table S3. Values of the harmonic mean of the ionization energies $\frac{\varepsilon_S \varepsilon_X}{(\varepsilon_S + \varepsilon_X)}$ of PMMA and organic solvents and the parameter $\frac{3\mathcal{N}}{2(4\pi\varepsilon_0)^2}\mathcal{P}_{S-X}$ for the various organic molecules.

**Molecule**	$\frac{\varepsilon_S \varepsilon_X}{(\varepsilon_S + \varepsilon_X)}$ (10^{-19}J)	$\frac{3\mathcal{N}}{2(4\pi\varepsilon_0)^2}\mathcal{P}_{S-X}$ (10^{-15} SI)
n-pentane	6.947	56.348
n-hexane	6.904	66.704
n-heptane	6.846	75.640
n-octane	6.807	87.865
n-octane	6.779	95.548
CCl_4	7.266	64.003
$CHCl_3$	7.228	42.309
CH_2Cl_2	7.241	52.112
Diethyl ether	6.718	51.648
Tetrahydrofuran	6.677	44.558
Ethyl acetate	6.869	51.084

Table S4. Values of ($-\Delta G_a^p(T)$ kJ/mol) of polar molecules adsorbed on silica particles as a function of the temperature.

**Polar free energy of solvents adsorbed on silica ($\theta = 0$)**							
Temperature T(K)	CCl_4	CH_2Cl_2	$CHCl_3$	Diethyl ether	THF	Ethyl acetate	Toluene
303.15	6.674	25.241	20.030	28.337	37.697	17.422	17.701
313.15	6.715	24.896	19.854	27.581	36.486	17.089	17.452
323.15	6.752	24.807	19.752	26.838	35.506	16.852	17.328
328.15	6.777	24.379	19.590	26.447	34.669	16.590	17.078
333.15	6.797	24.206	19.502	26.069	34.064	16.423	16.954
338.15	6.818	24.034	19.414	25.691	33.458	16.257	16.829
343.15	6.809	23.876	19.303	25.462	32.786	16.149	16.722
348.15	6.859	23.689	19.238	24.935	32.247	15.924	16.580

353.15	6.879	23.516	19.150	24.557	31.642	15.757	16.456
363.15	6.884	23.102	18.927	23.805	30.437	15.394	16.170
373.15	6.961	22.826	18.798	23.045	29.220	15.091	15.958
378.15	6.982	22.654	18.710	22.667	28.614	14.925	15.833
383.15	6.969	22.285	18.547	22.315	27.908	14.704	15.598
388.15	7.023	22.309	18.534	21.911	27.403	14.592	15.584
393.15	7.043	22.136	18.446	21.533	26.798	14.425	15.460
398.15	7.064	21.964	18.358	21.155	26.192	14.259	15.335
403.15	7.127	21.689	18.248	20.674	25.592	14.080	15.184
408.15	7.105	21.619	18.182	20.399	24.981	13.926	15.086
413.15	7.125	21.446	18.094	20.021	24.376	13.759	14.962
423.15	7.205	21.206	18.006	19.185	23.401	13.498	14.834
433.15	7.207	20.756	17.742	18.509	21.954	13.093	14.464
443.15	7.248	20.411	17.566	17.753	20.743	12.760	14.215
453.15	7.289	20.066	17.390	16.997	19.532	12.427	13.966
463.15	7.348	20.070	17.346	16.496	18.659	12.350	13.973
473.15	7.371	19.376	17.038	15.485	17.110	11.761	13.468

Table S5. Values of ($-\Delta G_a^p(T)$ kJ/mol) of polar molecules adsorbed on PMMA particles as a function of the temperature.

**Polar free energy of solvents adsorbed on PMMA**							
Temperature T(K)	CCl_4	CH_2Cl_2	$CHCl_3$	Diethyl ether	THF	Ethyl acetate	Toluene
303.15	10.765	18.520	16.079	15.039	19.851	16.029	13.204
313.15	10.254	16.940	14.930	13.956	18.940	14.694	12.212
323.15	10.294	16.034	15.101	14.437	18.822	14.037	11.443
328.15	10.808	15.902	15.552	15.362	19.155	14.498	11.677
333.15	11.434	17.105	16.068	16.544	19.846	15.192	12.190
338.15	9.631	14.792	13.161	13.037	17.712	13.243	10.501
343.15	10.629	15.055	14.098	13.685	18.247	13.912	11.245
348.15	11.414	15.178	15.013	14.115	18.667	14.430	11.989
353.15	11.721	14.911	14.782	13.980	18.464	14.285	11.936
363.15	10.816	13.717	12.961	12.873	17.352	13.499	10.680
373.15	10.821	12.279	9.454	11.020	16.585	12.891	10.111
378.15	11.206	13.019	10.825	10.962	17.252	13.325	10.604
383.15	11.772	14.521	12.968	12.321	18.652	14.659	12.181
388.15	12.487	10.832	12.193	10.936	16.403	11.700	9.624
393.15	10.950	12.707	11.533	11.870	15.718	10.781	8.900
398.15	11.029	12.170	10.930	11.397	15.638	12.783	10.237
403.15	11.465	11.776	10.791	11.399	15.942	13.202	10.136
408.15	11.830	11.631	10.794	11.360	16.258	13.460	10.355
413.15	11.758	11.135	10.040	10.942	16.106	13.146	10.197
423.15	11.929	11.458	10.113	11.090	17.047	13.434	10.433
433.15	13.579	13.574	11.582	12.569	19.030	15.576	12.048
443.15	12.136	10.220	8.929	9.901	15.065	13.114	10.016
453.15	12.047	9.388	7.939	9.244	14.165	12.620	9.682

463.15	12.299	8.917	7.617	9.151	13.848	12.645	9.287
473.15	11.924	7.776	6.642	8.470	12.938	11.811	8.280

Table S6. Values of ($-\Delta G_a^p(T)$ kJ/mol) of polar molecules adsorbed on the system PMMA/silica as a function of the temperature for a recovery fraction $\theta = 0.31$.

**Polar free energy of solvents adsorbed on PMMA/silica for $\theta = 0.31$**							
Temperature T(K)	CCl_4	CH_2Cl_2	$CHCl_3$	Diethyl ether	THF	Ethyl acetate	Toluene
303.15	6.698	20.777	15.865	16.603	20.647	14.300	14.620
313.15	6.055	19.604	14.885	15.493	18.449	12.803	13.694
323.15	5.932	18.973	14.390	14.799	17.228	12.216	13.726
328.15	5.704	18.169	13.853	14.282	16.141	11.486	13.530
333.15	6.253	18.442	14.047	14.603	16.362	11.672	14.064
338.15	6.519	18.608	14.218	13.973	16.216	11.637	14.235
343.15	6.605	19.382	14.562	13.340	15.835	11.926	14.005
348.15	6.730	18.336	12.890	12.001	14.743	10.618	13.470
353.15	7.264	18.364	13.507	12.453	14.008	10.503	13.290
363.15	6.952	18.115	13.879	11.315	12.626	9.424	13.254
373.15	6.584	17.019	12.869	9.437	10.630	7.416	12.282
378.15	6.844	17.200	13.082	8.936	10.315	7.107	12.492
383.15	6.714	16.865	12.933	8.706	9.823	6.584	12.327
388.15	7.111	17.412	13.320	9.047	10.139	6.829	12.940
393.15	7.605	18.243	13.964	9.984	10.797	7.413	13.547
398.15	8.913	19.648	15.146	11.268	12.232	8.909	14.775
403.15	11.229	21.423	17.010	11.072	12.576	8.802	16.772
408.15	10.654	19.980	15.734	11.281	11.759	7.117	15.461
413.15	9.879	18.756	14.746	8.496	9.743	7.482	12.252
423.15	8.281	17.133	13.433	6.152	6.444	7.003	12.634
433.15	8.092	16.598	13.170	6.621	7.368	7.619	13.247
443.15	12.550	21.255	17.518	8.654	8.665	11.397	16.279
453.15	10.367	18.117	14.996	10.447	8.862	11.040	14.673
463.15	8.354	15.912	12.490	10.442	1.559	9.852	11.573
473.15	7.697	12.895	10.572	9.325	0.595	8.197	8.969

Table S7. Values of ($-\Delta G_a^p(T)$ kJ/mol) of polar molecules adsorbed on the system PMMA/silica as a function of the temperature for a recovery fraction $\theta = 0.83$.

**Polar free energy of solvents adsorbed on PMMA/silica for $\theta = 0.83$**							
Temperature T(K)	CCl_4	CH_2Cl_2	$CHCl_3$	Diethyl ether	THF	Ethyl acetate	Toluene
303.15	7.773	27.605	23.019	21.816	25.479	20.114	17.258
313.15	7.219	25.780	21.588	20.382	24.106	18.354	15.812
323.15	7.726	24.330	20.823	19.402	23.091	16.800	15.052
328.15	7.983	24.125	20.151	19.158	22.906	16.021	14.985
333.15	8.322	24.143	20.132	18.742	22.962	15.604	15.539
338.15	8.421	24.970	20.394	18.368	23.217	16.923	15.672

343.15	7.970	23.919	20.547	18.073	21.957	16.634	14.982
348.15	8.213	23.804	20.465	17.719	21.787	16.379	15.316
353.15	8.480	23.752	20.339	17.763	21.690	16.315	15.442
363.15	7.946	22.566	19.802	16.558	19.764	14.651	13.973
373.15	7.400	21.338	18.643	15.259	18.336	12.867	13.259
378.15	7.177	21.114	18.505	14.790	17.504	12.511	12.966
383.15	8.088	21.872	19.412	15.216	18.162	13.086	13.592
388.15	9.738	23.872	20.633	17.249	20.357	14.790	15.021
393.15	7.975	22.237	18.677	16.071	19.312	12.425	13.092
398.15	7.579	21.453	18.849	14.284	17.365	9.449	10.478
403.15	8.266	21.428	19.153	13.895	17.001	11.597	13.409
408.15	8.378	20.973	18.729	13.601	16.463	11.654	13.423
413.15	8.176	20.481	18.274	13.057	15.661	11.281	13.111
423.15	8.263	20.132	18.597	12.392	14.863	11.137	12.497
433.15	9.011	21.753	19.964	13.615	16.245	12.413	13.101
443.15	8.413	19.670	18.315	11.083	13.556	10.859	12.321
453.15	8.373	19.017	17.896	10.411	12.693	10.113	12.024
463.15	11.973	22.320	21.465	13.227	15.364	12.954	15.267
473.15	8.067	17.745	16.842	8.413	10.414	7.945	10.717

Table S8. Values of ($-\Delta G_a^p(T)$ kJ/mol) of polar molecules adsorbed on the system PMMA/silica as a function of the temperature for a recovery fraction $\theta = 1$.

**Polar free energy of solvents adsorbed on PMMA/silica for $\theta = 1$**							
Temperature T(K)	CCl_4	CH_2Cl_2	$CHCl_3$	Diethyl ether	THF	Ethyl acetate	Toluene
303.15	13.797	36.031	32.260	30.623	38.590	21.479	26.098
313.15	13.567	31.827	28.141	27.132	33.996	20.360	23.011
323.15	12.594	27.832	24.874	23.581	28.575	18.376	20.293
328.15	12.366	26.052	22.043	21.509	26.236	17.682	17.852
333.15	12.572	25.107	21.512	20.173	24.834	17.746	17.145
338.15	11.175	23.004	19.957	18.075	22.883	17.019	15.721
343.15	7.827	22.303	19.418	17.043	22.426	16.908	15.316
348.15	8.731	23.918	21.243	17.929	23.166	17.644	16.472
353.15	9.682	24.387	21.614	18.557	23.527	17.500	17.214
363.15	11.294	24.438	22.228	19.161	23.921	16.971	17.656
373.15	11.607	23.218	21.724	18.422	22.395	15.990	16.949
378.15	11.599	23.350	21.692	18.202	22.230	16.213	17.043
383.15	12.922	25.912	23.757	19.957	24.657	18.321	18.608
388.15	14.514	28.129	25.061	21.098	23.208	20.016	17.299
393.15	11.630	25.838	23.488	18.299	21.089	17.162	14.154
398.15	9.405	22.887	20.451	15.594	19.094	15.325	12.647
403.15	7.366	18.964	17.081	12.048	17.162	14.041	13.030
408.15	7.781	18.878	17.221	12.010	16.629	13.365	13.403
413.15	8.249	19.192	17.691	12.004	16.478	13.009	13.687
423.15	9.622	19.875	18.807	12.975	17.161	13.139	14.195
433.15	8.548	17.697	16.005	9.842	15.043	14.366	12.628

443.15	5.562	14.849	14.031	7.101	11.750	12.211	9.983
453.15	6.883	15.433	14.892	7.366	11.880	12.355	11.138
463.15	8.179	16.389	16.175	8.339	12.549	12.883	11.825
473.15	8.812	16.525	16.477	8.298	12.238	12.504	11.793

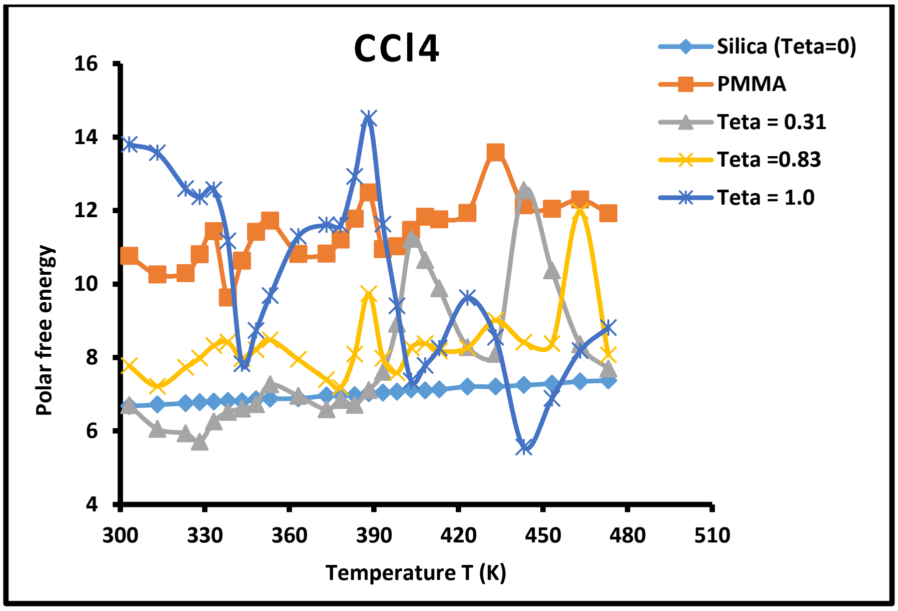

Figure S1. Variations of the polar free interaction energy of CCl4 adsorbed on PMMA/silica a function of the temperature, at different recovery fractions.

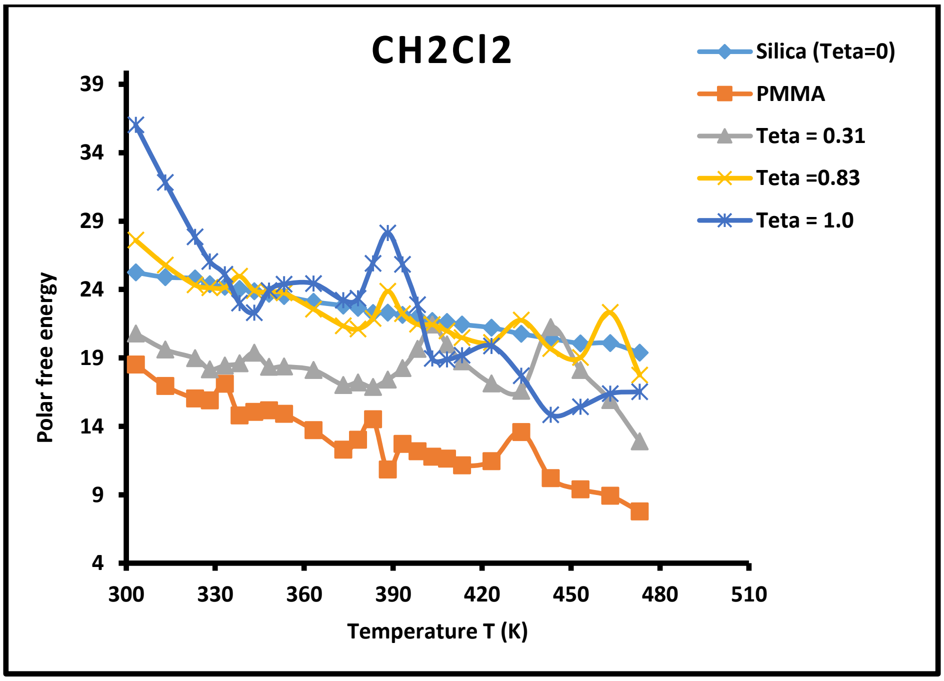

Figure S2. Variations of the polar free interaction energy of CH2Cl2 adsorbed on PMMA/silica a function of the temperature, at different recovery fractions.

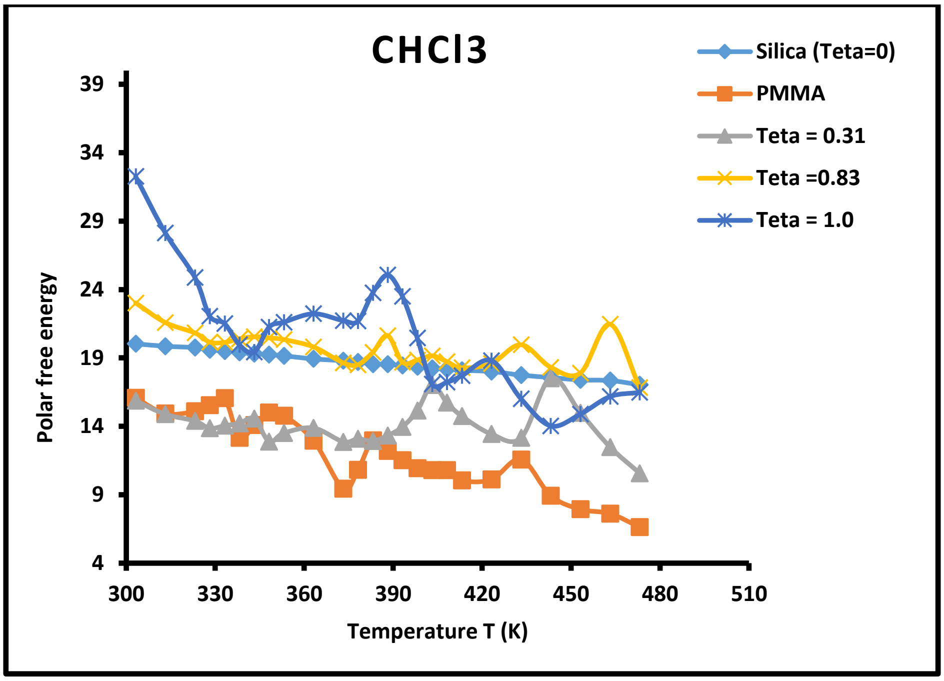

Figure S3. Variations of the polar free interaction energy of CHCl3 adsorbed on PMMA/silica as a function of the temperature, at different recovery fractions.

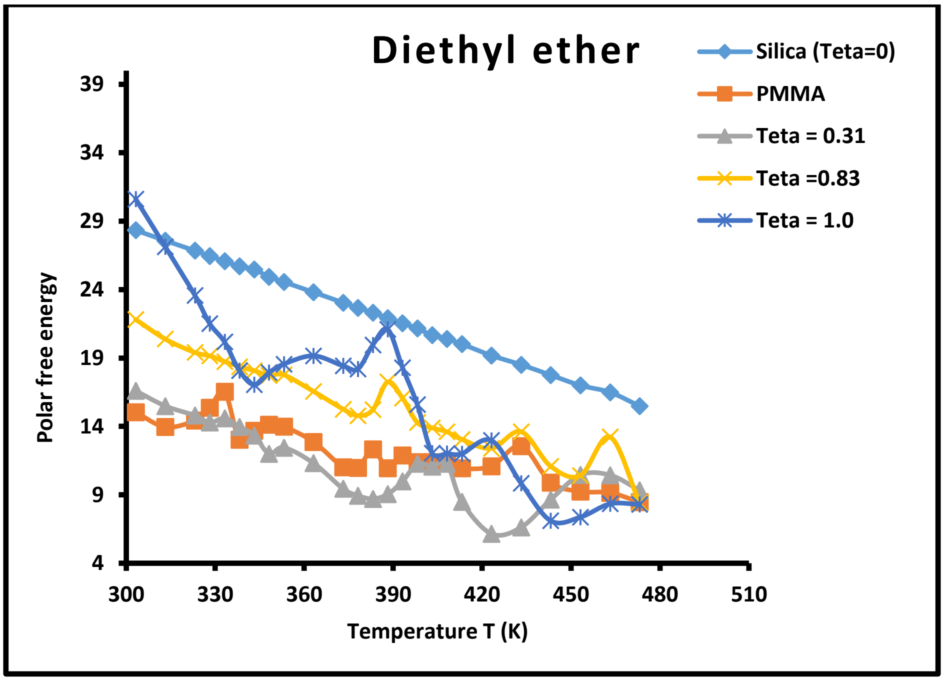

Figure S4. Variations of the polar free interaction energy of diethyl ether adsorbed on PMMA/silica as a function of the temperature, at different recovery fractions.

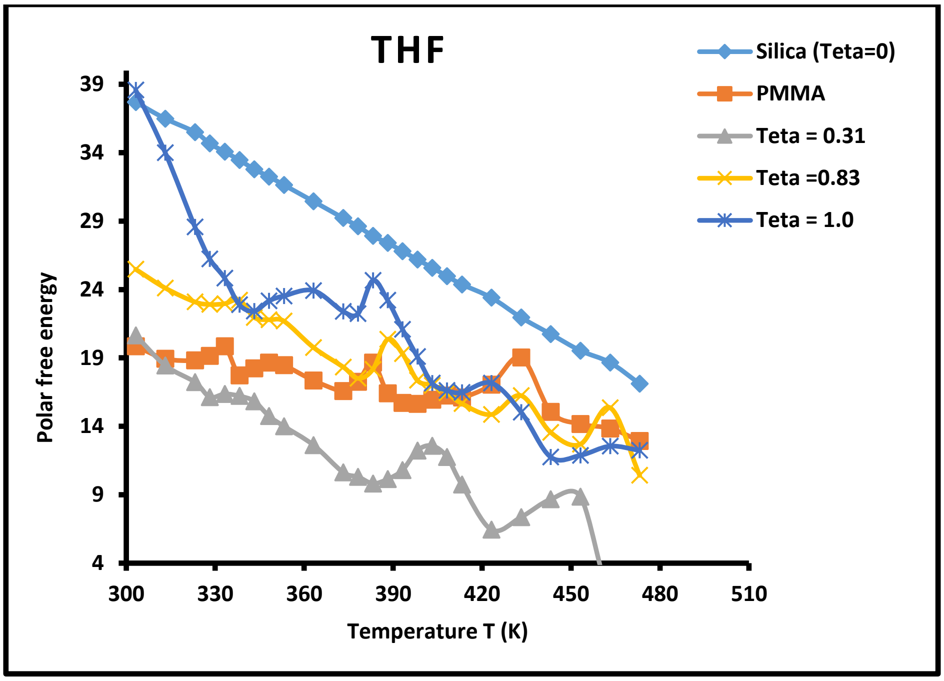

Figure S5. Variations of the polar free interaction energy of THF adsorbed on PMMA/silica as a function of the temperature, at different recovery fractions.

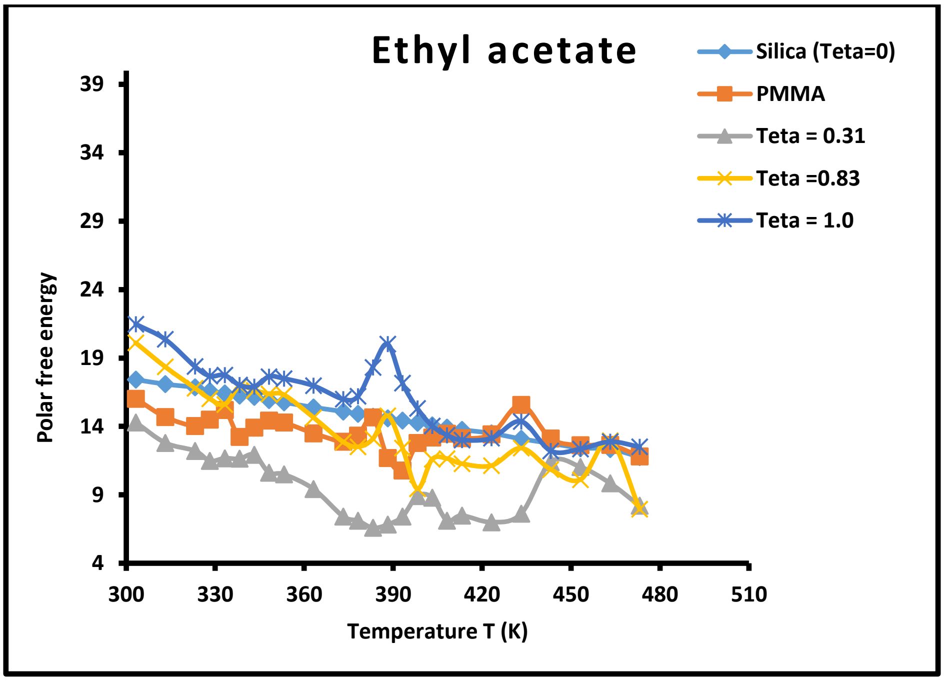

Figure S6. Variations of the polar free interaction energy of ethyl acetate adsorbed on PMMA/silica as a function of the temperature, at different recovery fractions.

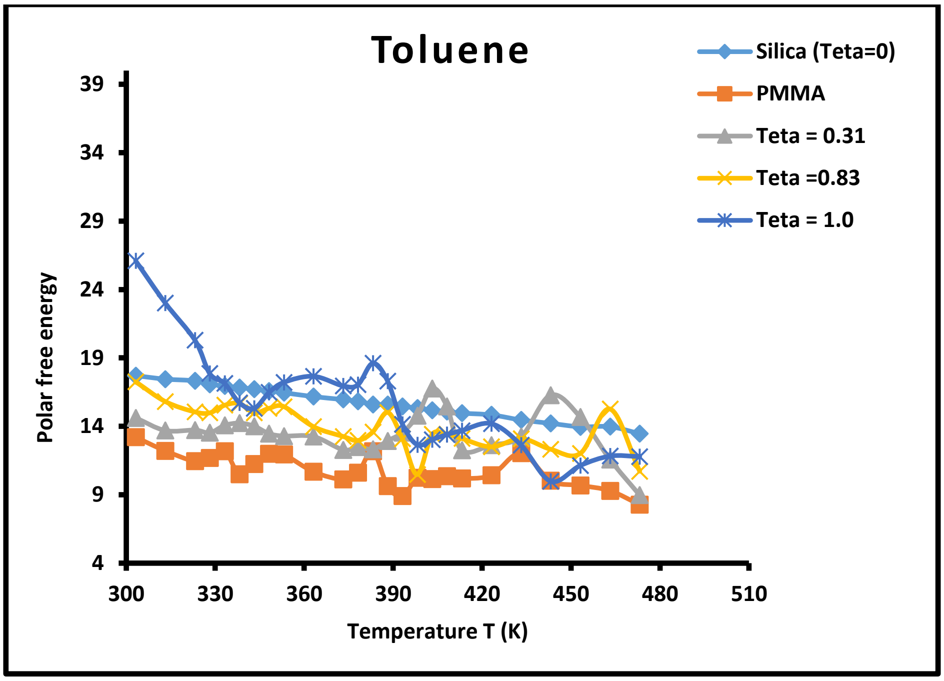

Figure S7. Variations of the polar free interaction energy of toluene adsorbed on PMMA/silica as a function of the temperature, at different recovery fractions.

Table S9. Values (in kJ/mol) of polar enthalpy $(-\Delta H_a^p(T)$ and $(-\Delta S_a^p(T)$ of polar solvents adsorbed on PMMA particles at different temperatures.

$(-\Delta H_a^p(T)$ (kJ/mol) of PMMA							
T(K)	CCl4	CH2Cl2	CHCl3	Diethyl ether	THF	Ethyl acetate	Toluene
303.15	43.650	96.121	66.440	76.111	62.070	85.500	64.250
313.15	18.382	53.596	34.393	27.423	31.872	47.290	37.133
323.15	-7.706	9.691	1.305	-22.845	0.693	7.839	9.136
328.15	-21.058	-12.779	-15.629	-48.571	-15.264	-12.351	-5.193
333.15	-34.615	-35.594	-32.822	-74.692	-31.466	-32.851	-19.741
338.15	-77.626	-18.584	-92.255	-51.103	-37.851	-49.305	-66.791
343.15	-47.308	-0.870	-51.377	-26.576	-14.687	-27.163	-36.133
348.15	-16.545	17.104	-9.899	-1.689	8.817	-4.695	-5.024
353.15	14.663	35.338	32.179	23.557	32.661	18.097	26.534
363.15	78.414	72.585	118.135	75.131	81.370	64.656	91.001
373.15	-9.177	-8.062	-62.810	72.882	-10.842	13.663	12.572
378.15	-22.701	-65.161	-120.660	-33.427	-66.062	-53.954	-68.944
383.15	-36.404	-123.020	-179.280	-141.151	-122.018	-122.471	-151.545
388.15	196.168	-233.768	70.944	-121.742	91.509	200.631	147.591
393.15	69.988	-45.474	66.646	-11.969	44.240	-27.508	-13.357
398.15	-57.807	145.229	62.294	99.208	-3.633	-258.568	-176.365
403.15	-41.190	8.609	-23.498	4.627	-25.119	-24.806	-25.926
408.15	-5.898	37.005	37.755	35.457	13.012	21.844	4.498
413.15	29.828	65.750	99.763	66.666	51.613	69.069	35.297
423.15	-117.633	-202.173	-139.848	-144.642	-200.139	-179.314	-141.682

433.15	15.093	31.597	36.550	32.612	54.182	17.635	14.164
443.15	150.920	270.826	217.068	214.006	314.443	219.184	173.651
453.15	12.047	9.388	7.939	9.244	14.165	12.620	9.682
463.15	12.299	8.917	7.617	9.151	13.848	12.645	9.287
473.15	11.924	7.776	6.642	8.470	12.938	11.811	8.280

$(-\Delta S_a^p(T)$ (J/(K mol) of PMMA							
T(K)	CCl4	CH2Cl2	CHCl3	Diethyl ether	THF	Ethyl acetate	Toluene
303.15	114.0	245.9	178.4	215.4	129.9	239.0	165.2
313.15	32.0	107.9	74.4	57.4	31.9	115.0	77.2
323.15	-50.0	-30.1	-29.6	-100.6	-66.1	-9.0	-10.8
328.15	-91.0	-99.1	-81.6	-179.6	-115.1	-71.0	-54.8
333.15	-132.0	-168.1	-133.6	-258.6	-164.1	-133.0	-98.8
338.15	-244.2	-109.1	-327.1	-204.7	-172.0	-197.1	-222.5
343.15	-155.2	-57.1	-207.1	-132.7	-104.0	-132.1	-132.5
348.15	-66.2	-5.1	-87.1	-60.7	-36.0	-67.1	-42.5
353.15	22.8	46.9	32.9	11.3	32.0	-2.1	47.5
363.15	200.8	150.9	272.9	155.3	168.0	127.9	227.5
373.15	-48.3	-36.8	-190.0	178.7	-86.6	1.6	3.3
378.15	-84.3	-188.8	-344.0	-104.3	-233.6	-178.4	-213.7
383.15	-120.3	-340.8	-498.0	-387.3	-380.6	-358.4	-430.7
388.15	477.5	-644.3	165.1	-356.0	189.6	497.1	360.4
393.15	154.5	-162.3	154.1	-75.0	68.6	-86.9	-51.6
398.15	-168.5	319.7	143.1	206.0	-52.4	-670.9	-463.6
403.15	-144.5	-9.8	-93.9	-3.4	-93.6	-79.8	-97.5
408.15	-57.5	60.2	57.1	72.6	0.4	35.2	-22.5
413.15	29.5	130.2	208.1	148.6	94.4	150.3	52.5
423.15	-292.4	-525.0	-356.2	-380.6	-530.9	-466.1	-375.3
433.15	17.6	21.0	55.8	33.4	63.1	-6.1	-11.3
443.15	327.6	567.0	467.8	447.4	657.1	453.9	352.7
453.15	-89.4	57.8	35.3	-51.9	40.2	-35.3	43.0
463.15	-27.4	125.8	101.3	6.1	100.2	50.7	105.0
473.15	34.6	193.8	167.3	64.1	160.2	136.7	167.0

Table S10. Values (in kJ/mol) of polar enthalpy $(-\Delta H_a^p(T)$ and $(-\Delta S_a^p(T)$ of polar solvents adsorbed on PMMA/silica particles at different temperatures for a recovery fraction of 31%.

$(-\Delta H_a^p(T)$ (kJ/mol) of PMMA/silica for $\theta = 0.31$							
T(K)	CCl4	CH2Cl2	CHCl3	Diethyl ether	THF	Ethyl acetate	Toluene
303.15	40.240	63.260	53.000	64.210	94.190	67.620	47.560
313.15	24.833	52.167	42.523	49.419	72.003	52.213	27.222
323.15	8.925	40.713	31.706	34.148	49.097	36.305	6.224
328.15	0.784	34.852	26.170	26.332	37.373	28.164	-4.522
333.15	-7.482	28.900	20.549	18.397	25.470	19.898	-15.433

338.15	-18.357	24.452	62.357	70.492	67.402	42.997	44.226
343.15	-12.907	26.496	45.666	61.635	67.334	45.723	36.391
348.15	-7.377	28.570	28.729	52.648	67.265	48.488	28.441
353.15	-1.766	30.674	11.547	43.531	67.195	51.293	20.376
363.15	9.695	34.972	-23.552	24.907	67.051	57.023	3.901
373.15	-28.621	-16.518	-23.265	59.479	23.503	27.026	-17.893
378.15	0.680	22.173	3.781	39.194	36.651	43.179	10.281
383.15	30.371	61.380	31.188	18.639	49.974	59.547	38.829
388.15	-0.265	-28.095	-11.267	-47.037	-6.937	-1.020	-7.689
393.15	-63.941	-73.020	-53.066	-73.992	-67.487	-72.118	-56.130
398.15	-128.432	-118.519	-95.401	-101.292	-128.813	-144.126	-105.190
403.15	49.990	142.208	124.926	-120.458	33.918	226.173	51.337
408.15	66.216	124.360	101.399	122.527	131.274	59.857	205.484
413.15	82.642	106.291	77.581	368.506	229.830	-108.510	361.531
423.15	120.203	141.446	113.708	22.464	-30.866	49.716	38.623
433.15	-78.459	-81.192	-84.097	-44.328	-47.136	-85.579	-64.989
443.15	-281.761	-309.030	-286.522	-112.679	-63.786	-224.034	-171.021
453.15	129.355	107.784	1339.890	-6.168	482.666	47.633	161.618
463.15	67.046	145.352	1366.463	45.144	192.199	68.708	138.710
473.15	3.378	183.741	1393.616	97.577	-104.608	90.243	115.303

$(-\Delta S_a^p(T)$ (J/(K mol) of PMMA/silica for $\theta = 0.31$

T(K)	CCl4	CH2Cl2	CHCl3	Diethyl ether	THF	Ethyl acetate	Toluene
303.15	125.7	143.1	129.6	168.8	230.2	186.3	99.5
313.15	75.7	107.1	95.6	120.8	158.2	136.3	33.5
323.15	25.6	71.1	61.6	72.8	86.2	86.2	-32.5
328.15	0.7	53.1	44.6	48.8	50.2	61.3	-65.5
333.15	-24.3	35.1	27.6	24.8	14.2	36.3	-98.5
338.15	-82.7	15.3	136.5	167.0	152.0	105.7	77.9
343.15	-66.7	21.3	87.5	141.0	151.8	113.7	54.9
348.15	-50.7	27.3	38.5	115.0	151.6	121.7	31.9
353.15	-34.7	33.3	-10.5	89.0	151.4	129.7	8.9
363.15	-2.7	45.3	-108.5	37.0	151.0	145.7	-37.1
373.15	-97.9	-91.9	-115.0	140.9	23.6	64.6	-82.2
378.15	-19.9	11.1	-43.0	86.9	58.5	107.6	-7.2
383.15	58.1	114.1	29.0	32.9	93.6	150.6	67.8
388.15	-20.7	-125.5	-51.2	-136.1	-33.6	-14.7	-46.8
393.15	-183.7	-240.5	-158.2	-205.1	-188.6	-196.7	-170.8
398.15	-346.7	-355.5	-265.2	-274.1	-343.6	-378.7	-294.8
403.15	97.3	288.8	251.6	-311.6	62.2	536.7	99.4
408.15	137.3	244.8	193.6	287.4	302.2	126.7	479.4
413.15	177.3	200.8	135.6	886.4	542.2	-283.3	859.4
423.15	277.8	275.2	217.5	46.0	-102.6	105.5	62.8
433.15	-186.2	-244.8	-244.5	-110.0	-140.6	-210.5	-179.2

443.15	-650.2	-764.8	-706.5	-266.0	-178.6	-526.5	-421.2
453.15	255.8	215.9	311.0	-18.2	1044.3	66.0	313.8
463.15	119.8	297.9	253.0	93.8	410.3	112.0	263.8
473.15	-16.2	379.9	195.0	205.8	-223.7	158.0	213.8

Table S11. Values (in kJ/mol) of polar enthalpy $(-\Delta H_a^p(T)$ and $(-\Delta S_a^p(T)$ of polar solvents adsorbed on PMMA/silica for $\theta = 0.83$ at different temperatures.

$(-\Delta H_a^p(T)$ (kJ/mol) of PMMA/silica for $\theta = 0.83$							
T(K)	CCl4	CH2Cl2	CHCl3	Diethyl ether	THF	Ethyl acetate	Toluene
303.15	30.470	103.020	66.140	66.760	84.220	77.910	83.240
313.15	13.214	77.752	53.814	55.050	65.115	68.666	53.042
323.15	-4.603	51.664	41.088	42.961	45.390	59.121	21.863
328.15	-13.721	38.312	34.575	36.773	35.294	54.236	5.906
333.15	-22.979	24.755	27.962	30.491	25.044	49.277	-10.296
338.15	-0.192	51.232	13.685	28.422	52.813	16.204	9.255
343.15	2.192	49.869	20.839	34.553	56.560	28.467	20.156
348.15	4.612	48.487	28.098	40.775	60.362	40.910	31.217
353.15	7.066	47.084	35.461	47.087	64.219	53.534	42.438
363.15	12.081	44.219	50.504	59.980	72.099	79.321	65.359
373.15	64.731	78.178	64.065	85.487	132.221	74.919	67.967
378.15	-20.541	4.551	-14.446	18.246	20.277	5.048	-1.153
383.15	-106.949	-70.057	-94.002	-49.890	-93.157	-65.753	-71.192
388.15	204.970	185.373	253.266	87.057	59.188	171.457	135.848
393.15	98.323	118.962	86.849	134.716	129.505	219.116	189.367
398.15	-9.689	51.702	-81.698	182.986	200.722	267.386	243.571
403.15	-7.961	53.616	49.769	28.460	52.399	-12.743	-5.556
408.15	17.594	56.455	52.203	48.742	73.898	22.143	20.812
413.15	43.465	59.330	54.667	69.275	95.662	57.459	47.504
423.15	-58.125	-129.765	-100.056	-114.149	-121.359	-94.606	-45.114
433.15	-0.753	28.650	29.246	46.836	53.326	26.989	13.971
443.15	57.959	190.766	161.567	211.580	232.091	151.423	74.435
453.15	-332.165	-307.410	-320.758	-280.024	-281.058	-306.479	-304.948
463.15	11.447	53.612	54.925	70.003	68.052	52.711	52.409
473.15	362.560	422.514	438.808	427.669	424.782	419.740	417.566
$(-\Delta S_a^p(T)$ (J/(K mol) of PMMA/silica for $\theta = 0.83$							
T(K)	CCl4	CH2Cl2	CHCl3	Diethyl ether	THF	Ethyl acetate	Toluene
303.15	87.5	254.6	128.3	140.3	206.5	183.9	225.1
313.15	31.5	172.6	88.3	102.3	144.5	153.9	127.1
323.15	-24.5	90.6	48.3	64.3	82.5	123.9	29.1
328.15	-52.5	49.6	28.3	45.3	51.5	108.9	-19.9
333.15	-80.5	8.6	8.3	26.3	20.5	93.9	-68.9
338.15	-40.4	63.6	-12.7	36.6	82.2	1.2	-10.3

343.15	-33.4	59.6	8.3	54.6	93.2	37.2	21.7
348.15	-26.4	55.6	29.3	72.6	104.2	73.2	53.7
353.15	-19.4	51.6	50.3	90.6	115.2	109.2	85.7
363.15	-5.4	43.6	92.3	126.6	137.2	181.2	149.7
373.15	149.0	-161.5	111.3	193.2	295.3	167.8	143.1
378.15	-78.0	34.5	-97.7	14.2	-2.7	-18.2	-40.9
383.15	-305.0	230.5	-306.7	-164.8	-300.7	-204.2	-224.9
388.15	517.0	419.9	594.6	185.0	81.4	395.2	305.3
393.15	244.0	249.9	168.6	307.0	261.4	517.2	442.3
398.15	-29.0	79.9	-257.4	429.0	441.4	639.2	579.3
403.15	-26.8	72.2	70.1	38.4	94.5	-66.3	-58.9
408.15	36.2	79.2	76.1	88.4	147.5	19.7	6.1
413.15	99.2	86.2	82.1	138.4	200.5	105.7	71.1
423.15	-171.5	-361.5	-272.9	-288.6	-302.5	-230.5	-142.8
433.15	-37.5	8.5	29.1	87.4	105.5	53.5	-4.8
443.15	96.5	378.5	331.1	463.4	513.5	337.5	133.2
453.15	-767.8	-716.8	-728.7	-619.3	-649.0	-721.0	-685.3
463.15	-17.7	71.2	91.3	144.7	113.0	63.0	94.7
473.15	732.3	859.2	911.3	908.7	875.0	847.0	874.7

Table S12. Values (in kJ/mol) of polar enthalpy $(-\Delta H_a^p(T)$ and $(-\Delta S_a^p(T)$ of polar solvents adsorbed on PMMA/silica for $\theta = 1.0$ (monolayer) at different temperatures.

	$(-\Delta H_a^p(T)$ (kJ/mol) of PMMA/silica for $\theta = 1.0$ (monolayer)						
T(K)	CCl4	CH2Cl2	CHCl3	Diethyl ether	THF	Ethyl acetate	Toluene
303.15	43.650	96.121	66.440	76.111	62.070	85.500	64.250
313.15	18.382	53.596	34.393	27.423	31.872	47.290	37.133
323.15	-7.706	9.691	1.305	-22.845	0.693	7.839	9.136
328.15	-21.058	-12.779	-15.629	-48.571	-15.264	-12.351	-5.193
333.15	-34.615	-35.594	-32.822	-74.692	-31.466	-32.851	-19.741
338.15	-77.626	-18.584	-92.255	-51.103	-37.851	-49.305	-66.791
343.15	-47.308	-0.870	-51.377	-26.576	-14.687	-27.163	-36.133
348.15	-16.545	17.104	-9.899	-1.689	8.817	-4.695	-5.024
353.15	14.663	35.338	32.179	23.557	32.661	18.097	26.534
363.15	78.414	72.585	118.135	75.131	81.370	64.656	91.001
373.15	-9.177	-8.062	-62.810	72.882	-10.842	13.663	12.572
378.15	-22.701	-65.161	-120.660	-33.427	-66.062	-53.954	-68.944
383.15	-36.404	-123.020	-179.280	-141.151	-122.018	-122.471	-151.545
388.15	196.168	-233.768	70.944	-121.742	91.509	200.631	147.591
393.15	69.988	-45.474	66.646	-11.969	44.240	-27.508	-13.357
398.15	-57.807	145.229	62.294	99.208	-3.633	-258.568	-176.365
403.15	-41.190	8.609	-23.498	4.627	-25.119	-24.806	-25.926
408.15	-5.898	37.005	37.755	35.457	13.012	21.844	4.498

413.15	29.828	65.750	99.763	66.666	51.613	69.069	35.297
423.15	-117.633	-202.173	-139.848	-144.642	-200.139	-179.314	-141.682
433.15	15.093	31.597	36.550	32.612	54.182	17.635	14.164
443.15	150.920	270.826	217.068	214.006	314.443	219.184	173.651
453.15	12.047	9.388	7.939	9.244	14.165	12.620	9.682
463.15	12.299	8.917	7.617	9.151	13.848	12.645	9.287
473.15	11.924	7.776	6.642	8.470	12.938	11.811	8.280

$(-\Delta S_a^p(T)$ (J/(K mol) of PMMA/silica for $\theta = 1.0$ (monolayer)							
T(K)	CCl4	CH2Cl2	CHCl3	Diethyl ether	THF	Ethyl acetate	Toluene
303.15	114.0	245.9	178.4	215.4	129.9	239.0	165.2
313.15	32.0	107.9	74.4	57.4	31.9	115.0	77.2
323.15	-50.0	-30.1	-29.6	-100.6	-66.1	-9.0	-10.8
328.15	-91.0	-99.1	-81.6	-179.6	-115.1	-71.0	-54.8
333.15	-132.0	-168.1	-133.6	-258.6	-164.1	-133.0	-98.8
338.15	-244.2	-109.1	-327.1	-204.7	-172.0	-197.1	-222.5
343.15	-155.2	-57.1	-207.1	-132.7	-104.0	-132.1	-132.5
348.15	-66.2	-5.1	-87.1	-60.7	-36.0	-67.1	-42.5
353.15	22.8	46.9	32.9	11.3	32.0	-2.1	47.5
363.15	200.8	150.9	272.9	155.3	168.0	127.9	227.5
373.15	-48.3	-36.8	-190.0	178.7	-86.6	1.6	3.3
378.15	-84.3	-188.8	-344.0	-104.3	-233.6	-178.4	-213.7
383.15	-120.3	-340.8	-498.0	-387.3	-380.6	-358.4	-430.7
388.15	477.5	-644.3	165.1	-356.0	189.6	497.1	360.4
393.15	154.5	-162.3	154.1	-75.0	68.6	-86.9	-51.6
398.15	-168.5	319.7	143.1	206.0	-52.4	-670.9	-463.6
403.15	-144.5	-9.8	-93.9	-3.4	-93.6	-79.8	-97.5
408.15	-57.5	60.2	57.1	72.6	0.4	35.2	-22.5
413.15	29.5	130.2	208.1	148.6	94.4	150.3	52.5
423.15	-292.4	-525.0	-356.2	-380.6	-530.9	-466.1	-375.3
433.15	17.6	21.0	55.8	33.4	63.1	-6.1	-11.3
443.15	327.6	567.0	467.8	447.4	657.1	453.9	352.7
453.15	-89.4	57.8	35.3	-51.9	40.2	-35.3	43.0
463.15	-27.4	125.8	101.3	6.1	100.2	50.7	105.0
473.15	34.6	193.8	167.3	64.1	160.2	136.7	167.0

Table S13. Values (in kJ/mol) of polar enthalpy $(-\Delta H_a^p(T)$ and $(-\Delta S_a^p(T)$ of polar solvents adsorbed on silica particles at different temperatures.

$(-\Delta H_a^p(T)$ (kJ/mol) of silica							
T(K)	CCl4	CH2Cl2	CHCl3	Diethyl ether	THF	Ethyl acetate	Toluene
303.15	35.700	25.365	51.255	74.408	27.517	25.249	35.700
313.15	35.700	25.365	51.255	74.408	27.517	25.249	35.700
323.15	35.700	25.365	51.255	74.408	27.517	25.249	35.700

328.15	35.700	25.365	51.255	74.408	27.517	25.249	35.700
333.15	35.700	25.365	51.255	74.408	27.517	25.249	35.700
338.15	35.700	25.365	51.255	74.408	27.517	25.249	35.700
343.15	35.700	25.365	51.255	74.408	27.517	25.249	35.700
348.15	35.700	25.365	51.255	74.408	27.517	25.249	35.700
353.15	35.700	25.365	51.255	74.408	27.517	25.249	35.700
363.15	35.700	25.365	51.255	74.408	27.517	25.249	35.700
373.15	35.700	25.365	51.255	74.408	27.517	25.249	35.700
378.15	35.700	25.365	51.255	74.408	27.517	25.249	35.700
383.15	35.700	25.365	51.255	74.408	27.517	25.249	35.700
388.15	35.700	25.365	51.255	74.408	27.517	25.249	35.700
393.15	35.700	25.365	51.255	74.408	27.517	25.249	35.700
398.15	35.700	25.365	51.255	74.408	27.517	25.249	35.700
403.15	35.700	25.365	51.255	74.408	27.517	25.249	35.700
408.15	35.700	25.365	51.255	74.408	27.517	25.249	35.700
413.15	35.700	25.365	51.255	74.408	27.517	25.249	35.700
423.15	35.700	25.365	51.255	74.408	27.517	25.249	35.700
433.15	35.700	25.365	51.255	74.408	27.517	25.249	35.700
443.15	35.700	25.365	51.255	74.408	27.517	25.249	35.700
453.15	35.700	25.365	51.255	74.408	27.517	25.249	35.700
463.15	35.700	25.365	51.255	74.408	27.517	25.249	35.700
473.15	35.700	25.365	51.255	74.408	27.517	25.249	35.700

$(-\Delta S_a^p(T)$ (J/(K mol) of silica							
T(K)	CCl4	CH2Cl2	CHCl3	Diethyl ether	THF	Ethyl acetate	Toluene
303.15	-4.1	34.5	17.6	75.6	121.1	33.3	24.9
313.15	-4.1	34.5	17.6	75.6	121.1	33.3	24.9
323.15	-4.1	34.5	17.6	75.6	121.1	33.3	24.9
328.15	-4.1	34.5	17.6	75.6	121.1	33.3	24.9
333.15	-4.1	34.5	17.6	75.6	121.1	33.3	24.9
338.15	-4.1	34.5	17.6	75.6	121.1	33.3	24.9
343.15	-4.1	34.5	17.6	75.6	121.1	33.3	24.9
348.15	-4.1	34.5	17.6	75.6	121.1	33.3	24.9
353.15	-4.1	34.5	17.6	75.6	121.1	33.3	24.9
363.15	-4.1	34.5	17.6	75.6	121.1	33.3	24.9
373.15	-4.1	34.5	17.6	75.6	121.1	33.3	24.9
378.15	-4.1	34.5	17.6	75.6	121.1	33.3	24.9
383.15	-4.1	34.5	17.6	75.6	121.1	33.3	24.9
388.15	-4.1	34.5	17.6	75.6	121.1	33.3	24.9
393.15	-4.1	34.5	17.6	75.6	121.1	33.3	24.9
398.15	-4.1	34.5	17.6	75.6	121.1	33.3	24.9
403.15	-4.1	34.5	17.6	75.6	121.1	33.3	24.9
408.15	-4.1	34.5	17.6	75.6	121.1	33.3	24.9
413.15	-4.1	34.5	17.6	75.6	121.1	33.3	24.9

423.15	-4.1	34.5	17.6	75.6	121.1	33.3	24.9
433.15	-4.1	34.5	17.6	75.6	121.1	33.3	24.9
443.15	-4.1	34.5	17.6	75.6	121.1	33.3	24.9
453.15	-4.1	34.5	17.6	75.6	121.1	33.3	24.9
463.15	-4.1	34.5	17.6	75.6	121.1	33.3	24.9
473.15	-4.1	34.5	17.6	75.6	121.1	33.3	24.9

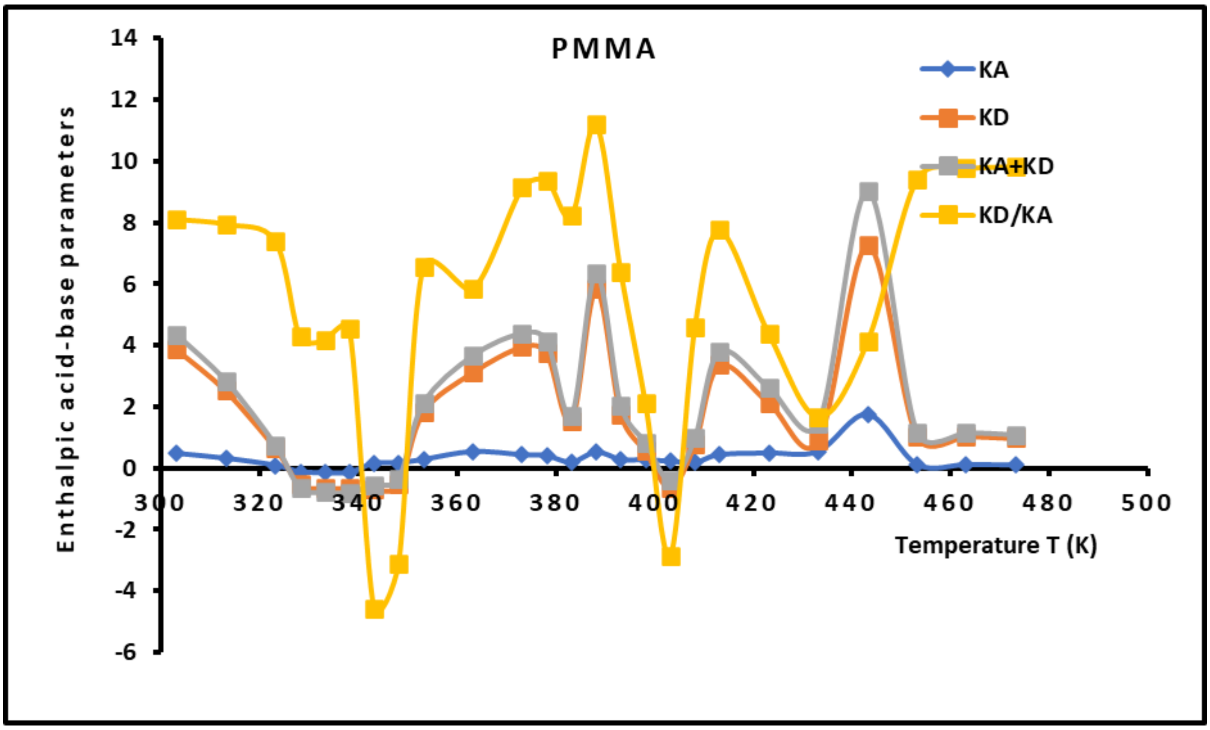

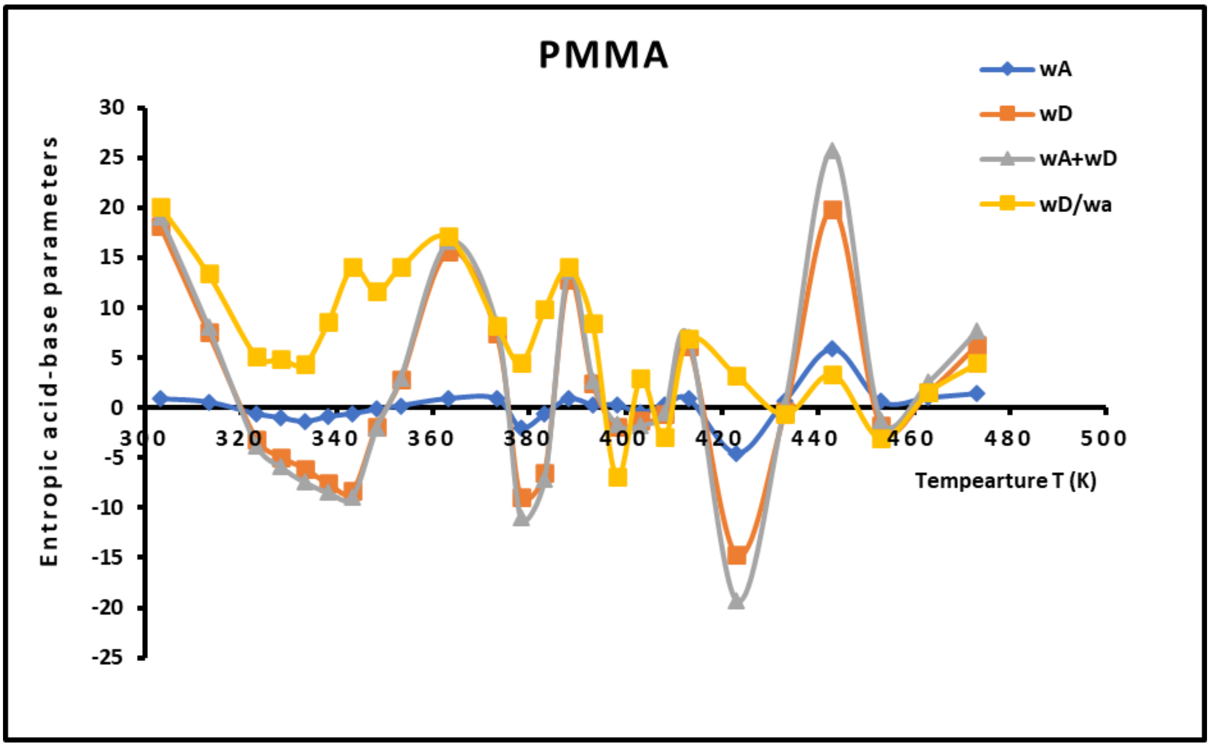

Figure S8. Evolutions of the enthalpic acid base parameters K_A, K_D, K_D/K_A and $K_D + K_A$, and the entropic acid base parameters ω_A , ω_D, ω_D / ω_A and $(\omega_D + \omega_A)$ of PMMA as a function of the temperature.

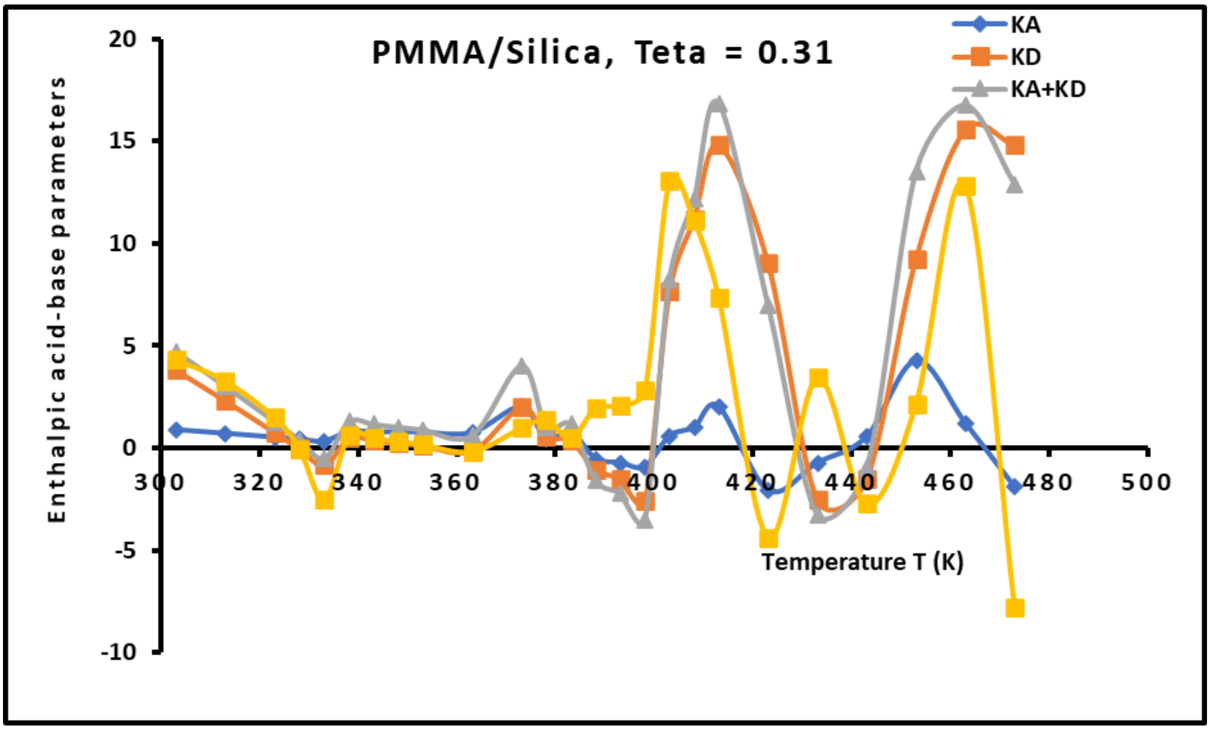

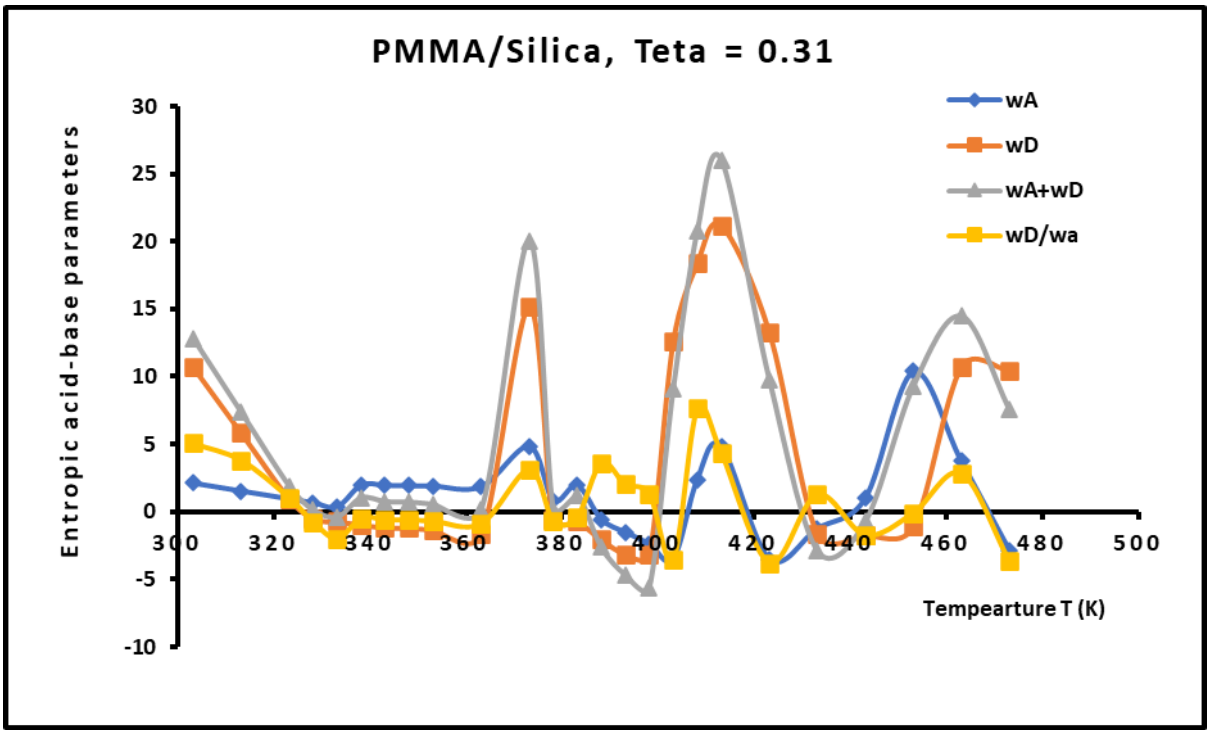

Figure S9. Evolutions of the enthalpic acid base parameters K_A, K_D, K_D/K_A and $K_D + K_A$, and the entropic acid base parameters ω_A , ω_D, ω_D / ω_A and $(\omega_D + \omega_A)$ of PMMA/silica for $\theta = 0.31$ as a function of the temperature.

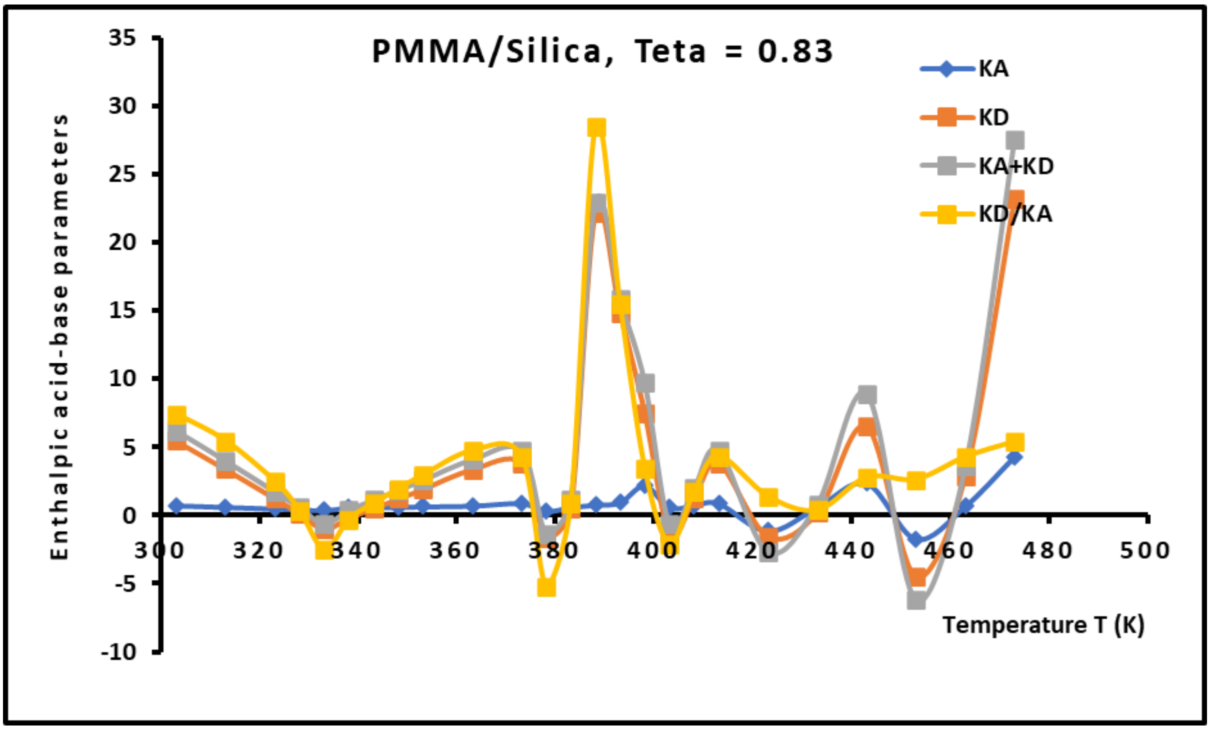

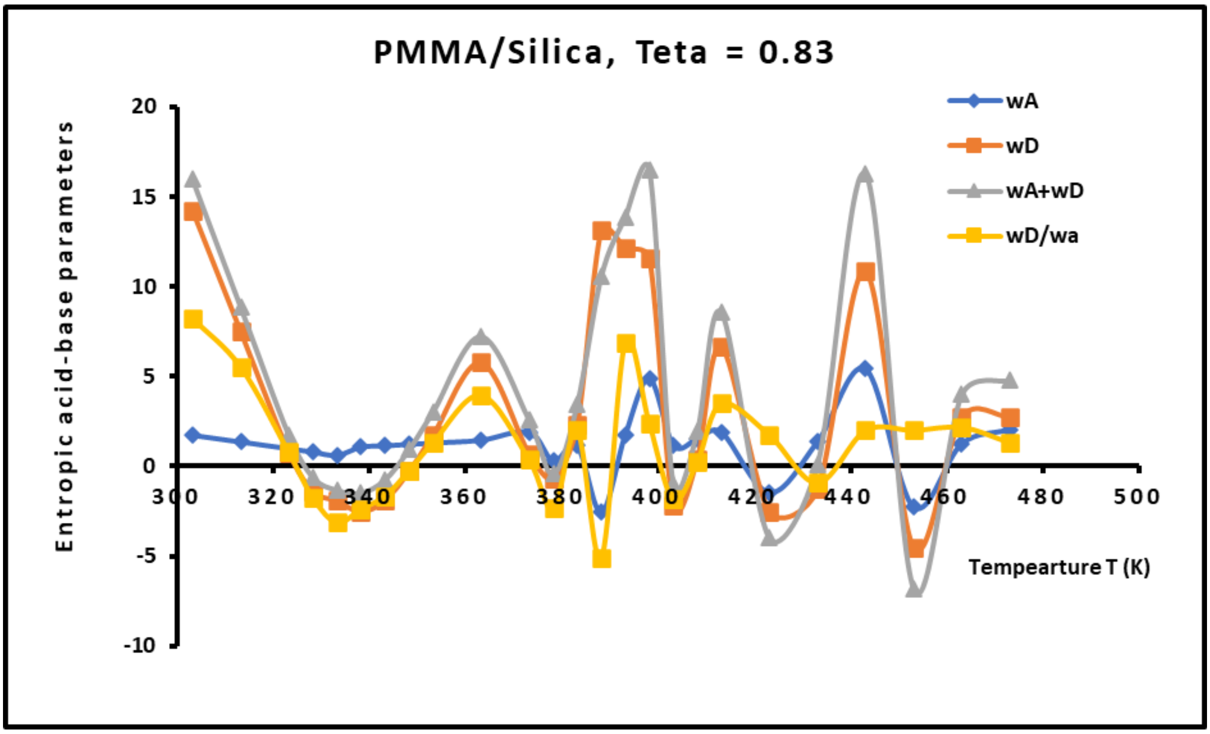

Figure S10. Evolutions of the enthalpic acid base parameters K_A, K_D, K_D/K_A and $K_D + K_A$, and the entropic acid base parameters ω_A , ω_D, ω_D / ω_A and $(\omega_D + \omega_A)$ of PMMA/silica for $\theta = 0.83$ as a function of the temperature.

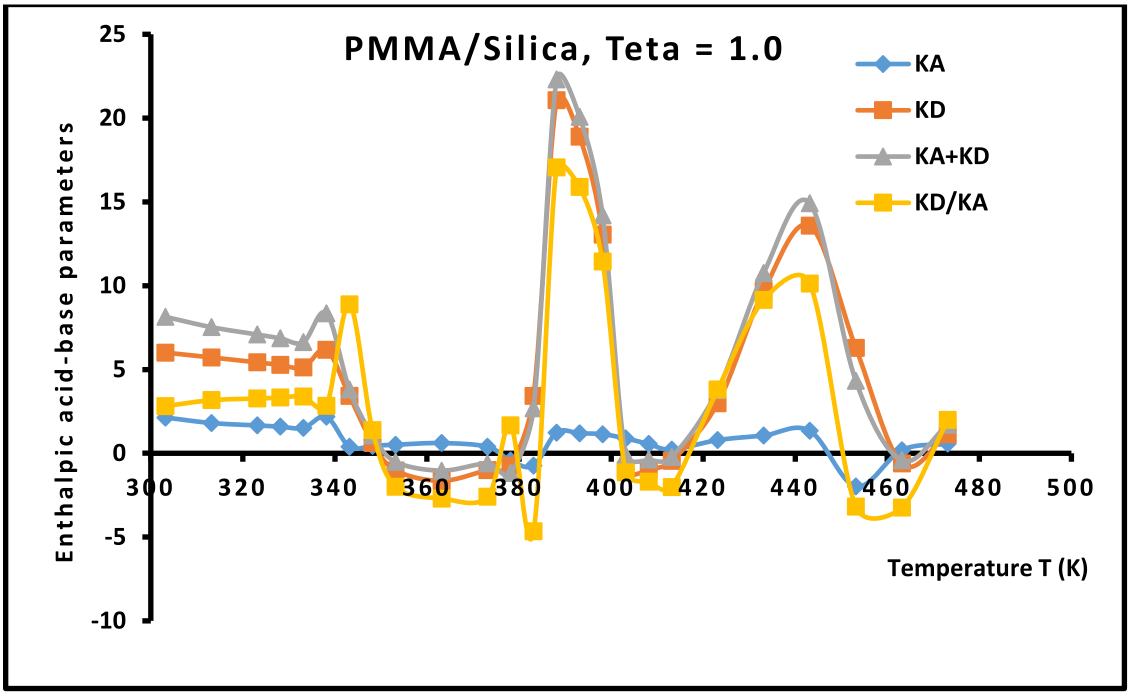

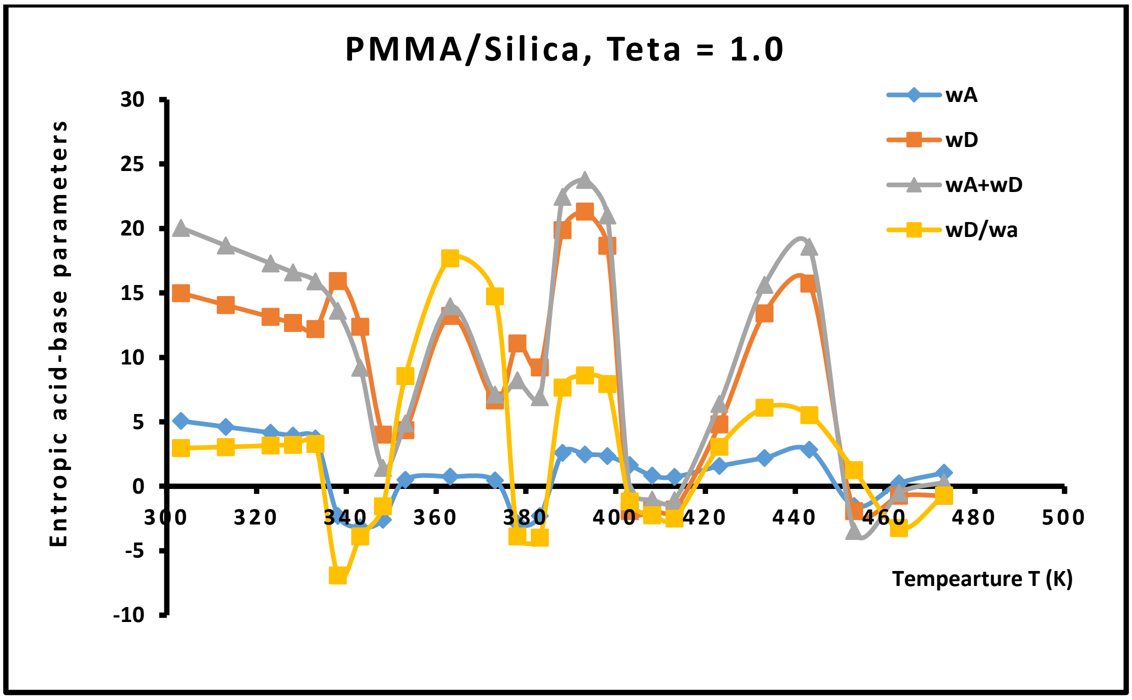

Figure S11. Evolutions of the enthalpic acid base parameters K_A, K_D, K_D/K_A and $K_D + K_A$, and the entropic acid base parameters ω_A , ω_D, ω_D / ω_A and $(\omega_D + \omega_A)$ of PMMA/silica for θ = 1.0 as a function of the temperature.

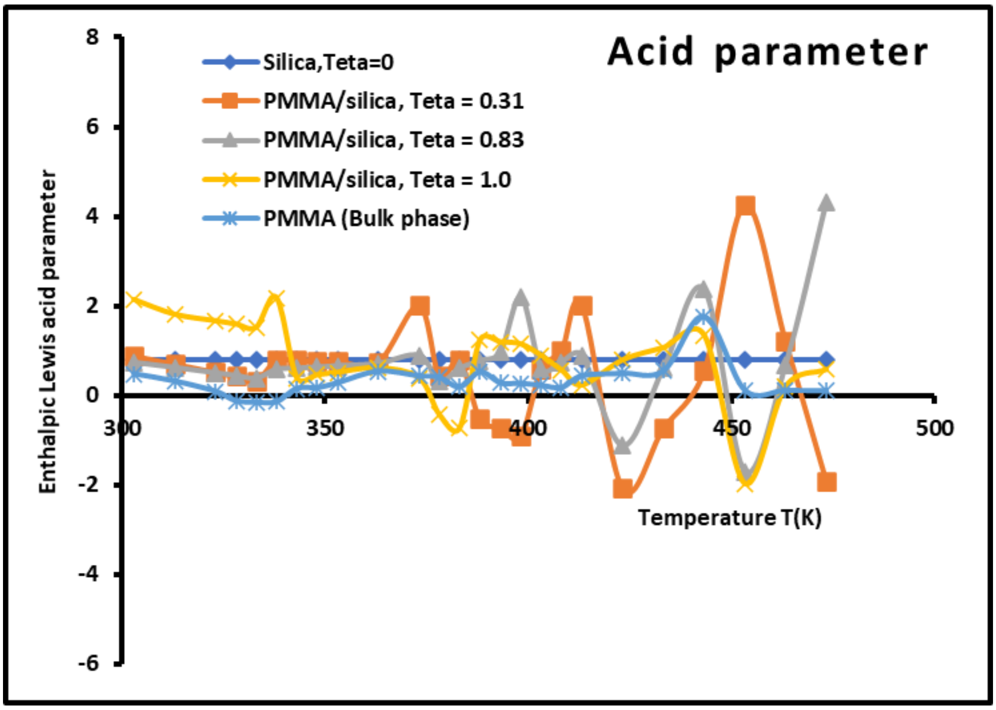

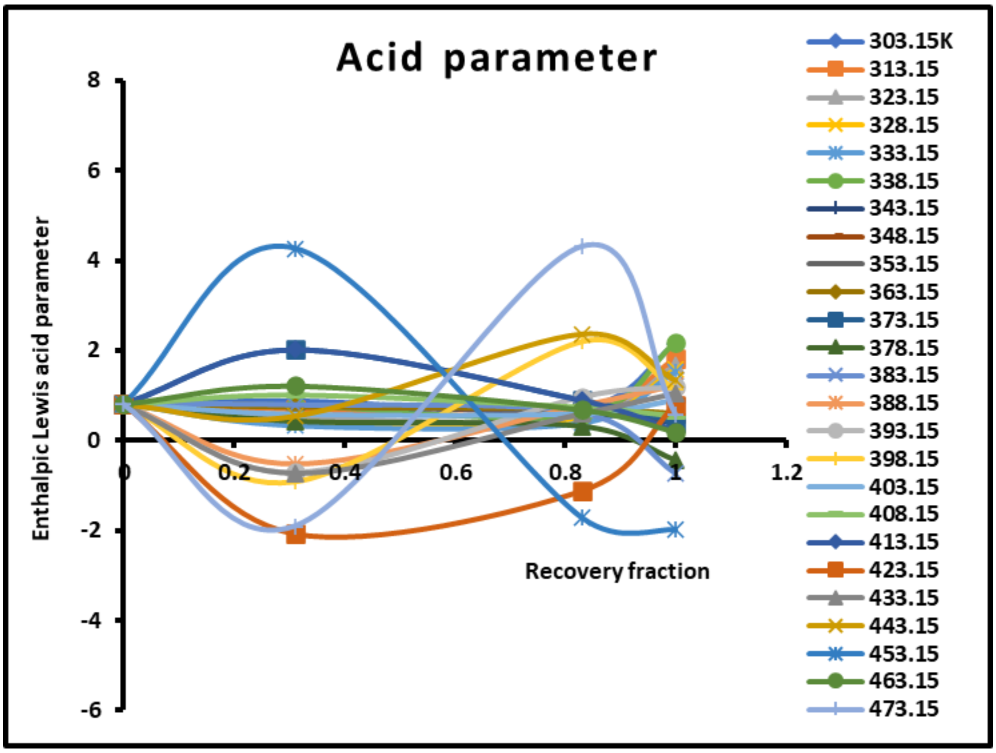

Figure S12. Evolutions of the enthalpic Lewis acid parameter K_A as a function of the recovery fraction and temperature.

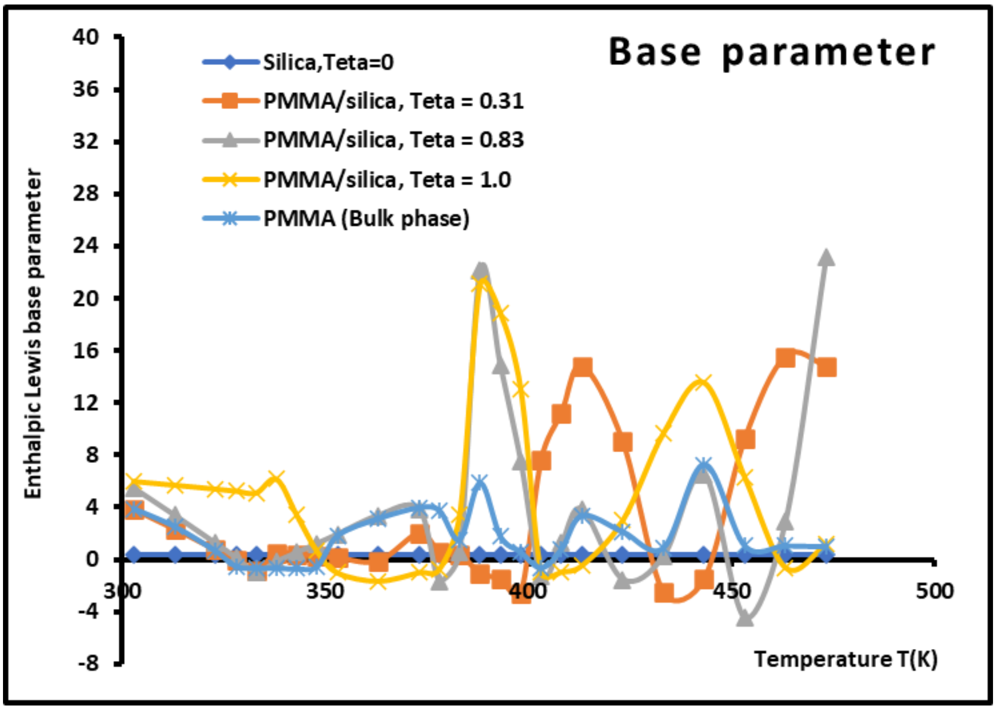

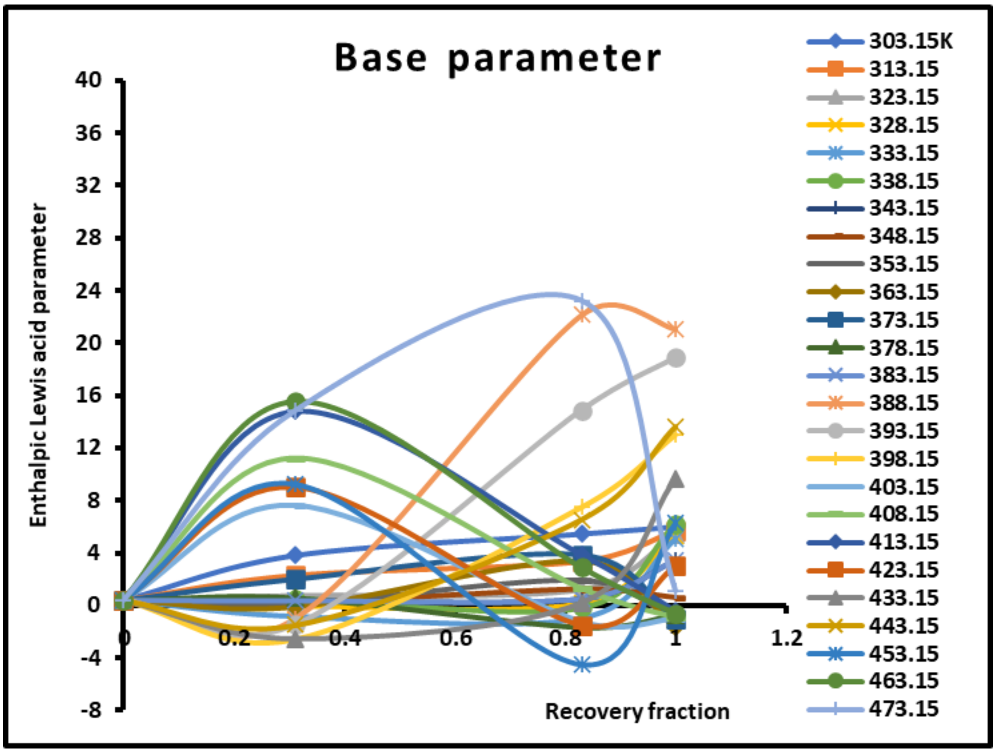

Figure S13. Evolutions of the enthalpic Lewis basic parameter K_D as a function of the recovery fraction and temperature.

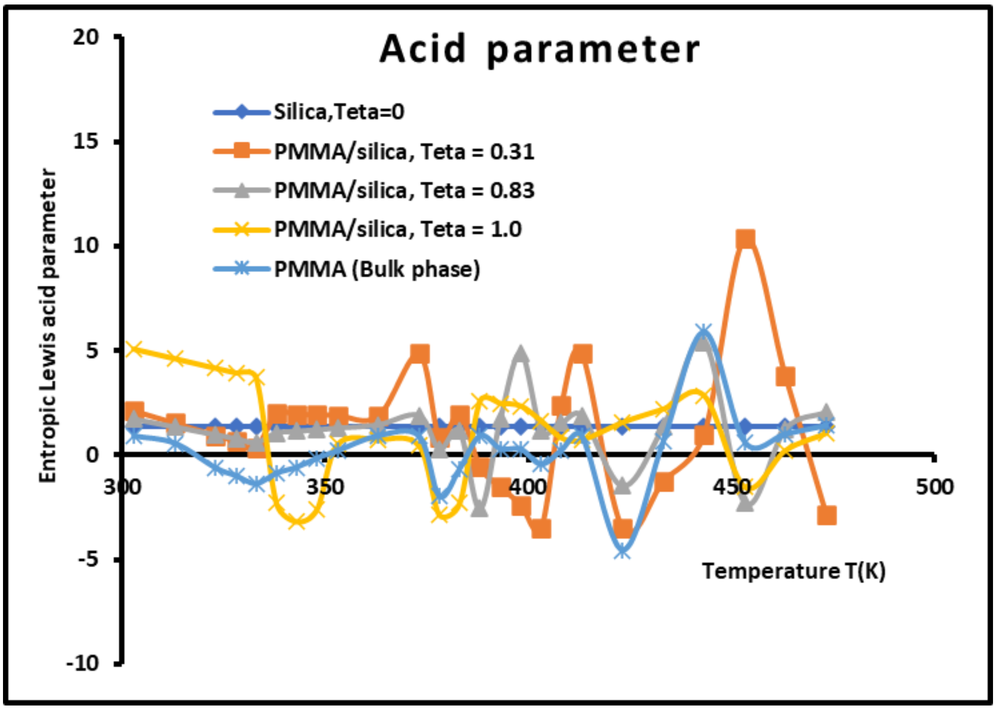

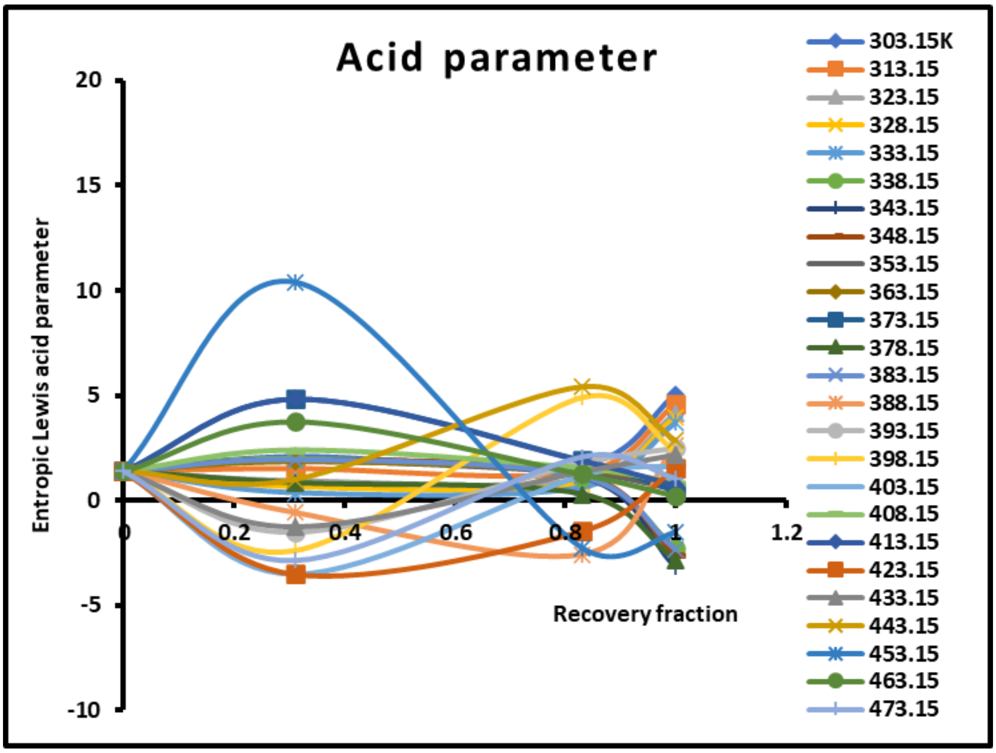

Figure S14. Evolutions of the entropic Lewis acidic parameter ω_A as a function of the recovery fraction and temperature.

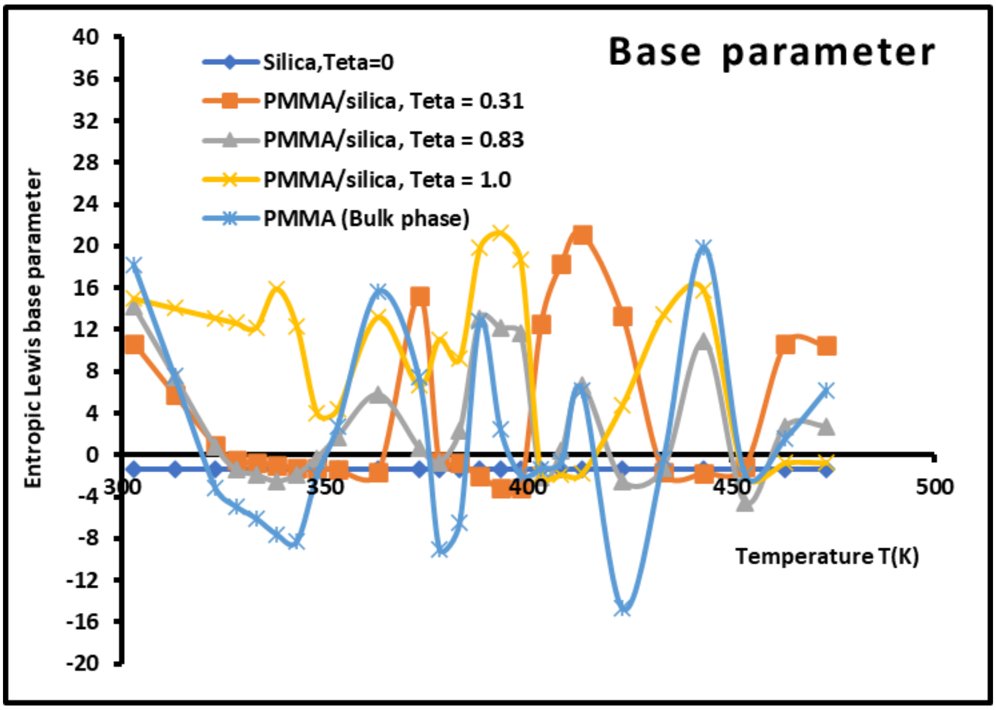

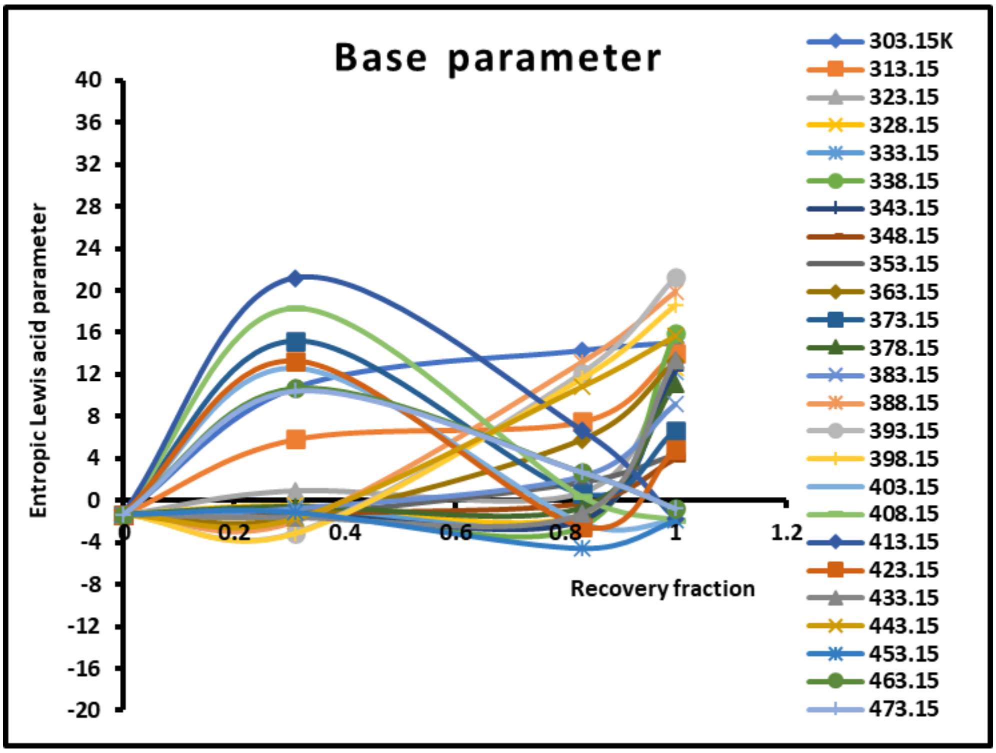

Figure S15. Evolutions of the entropic Lewis basic parameter ω_D as a function of the recovery fraction and temperature.

Table S14. Values (in kJ/mol) of the London free dispersive interaction energy $(-\Delta G_a^d(T)$ of organic solvents adsorbed on PMMA at different temperatures.

T(K)	C5	C6	C7	C8	C9	CCl4	CH2Cl2	CHCl3	Diethyl	THF	Ethyl	Toluene

								ether		acetate	
303.15	16.739	17.900	20.298	23.579	25.640	17.175	11.354	13.984	13.860	11.957	13.708	16.700
313.15	15.577	17.162	19.461	22.606	24.583	16.467	10.885	13.407	13.288	11.464	13.143	16.011
323.15	15.121	15.142	17.170	19.945	21.689	14.529	9.604	11.830	11.724	10.115	11.596	14.127
328.15	15.475	20.498	23.244	27.001	29.362	19.668	13.002	16.014	15.872	13.693	15.698	19.124
333.15	16.688	13.326	15.111	17.554	19.089	12.787	8.453	10.411	10.318	8.902	10.206	12.433
338.15	15.362	14.396	16.325	18.963	20.621	13.813	9.131	11.247	11.147	9.617	11.025	13.431
343.15	14.497	13.059	14.808	17.201	18.706	12.530	8.283	10.202	10.111	8.723	10.001	12.183
348.15	13.856	10.575	11.991	13.929	15.147	10.146	6.707	8.261	8.188	7.064	8.098	9.866
353.15	13.389	15.850	17.974	20.878	22.704	15.208	10.054	12.383	12.273	10.588	12.139	14.788
363.15	12.791	15.142	17.170	19.945	21.689	14.529	9.604	11.830	11.724	10.115	11.596	14.127
373.15	13.038	15.435	17.502	20.331	22.109	14.810	9.790	12.058	11.951	10.310	11.820	14.400
378.15	14.781	17.497	19.841	23.048	25.063	16.789	11.098	13.670	13.548	11.688	13.400	16.324
383.15	17.316	20.498	23.244	27.001	29.362	19.668	13.002	16.014	15.872	13.693	15.698	19.124
388.15	14.205	16.815	19.068	22.150	24.087	16.135	10.666	13.137	13.020	11.233	12.878	15.688
393.15	12.495	14.792	16.773	19.484	21.188	14.193	9.382	11.556	11.453	9.881	11.328	13.800
398.15	11.805	13.975	15.847	18.409	20.018	13.409	8.864	10.918	10.821	9.335	10.703	13.038
403.15	11.257	13.326	15.111	17.554	19.089	12.787	8.453	10.411	10.318	8.902	10.206	12.433
408.15	11.053	13.085	14.838	17.236	18.743	12.555	8.299	10.222	10.131	8.741	10.021	12.207
413.15	10.890	12.892	14.619	16.982	18.467	12.370	8.177	10.072	9.982	8.612	9.873	12.028
423.15	12.161	14.396	16.325	18.963	20.621	13.813	9.131	11.247	11.147	9.617	11.025	13.431
433.15	14.559	17.234	19.543	22.702	24.687	16.537	10.931	13.464	13.344	11.513	13.199	16.079
443.15	11.031	13.059	14.808	17.201	18.706	12.530	8.283	10.202	10.111	8.723	10.001	12.183
453.15	9.386	11.112	12.600	14.637	15.916	10.662	7.048	8.681	8.604	7.423	8.510	10.367
463.15	8.933	10.575	11.991	13.929	15.147	10.146	6.707	8.261	8.188	7.064	8.098	9.866
473.15	7.692	9.106	10.326	11.994	13.043	8.737	5.776	7.114	7.051	6.083	6.974	8.495

Table S15. Values (in kJ/mol) of the London free dispersive interaction energy ($-\Delta G_a^d(T)$ of organic solvents adsorbed on PMMA/silica for $\theta = 0.31$ at different temperatures.

T(K)	C5	C6	C7	C8	C9	CCl4	CH2Cl2	CHCl3	Diethyl ether	THF	Ethyl acetate	Toluene
303.15	22.286	26.382	29.916	34.751	37.790	25.314	16.734	20.611	20.427	17.623	20.204	24.613
313.15	20.936	24.784	28.104	32.646	35.501	23.780	15.720	19.362	19.190	16.556	18.980	23.122
323.15	19.947	23.612	26.776	31.103	33.823	22.657	14.977	18.447	18.283	15.773	18.083	22.030
328.15	19.637	23.246	26.360	30.620	33.298	22.305	14.744	18.161	17.999	15.528	17.802	21.687
333.15	19.891	23.547	26.702	31.017	33.730	22.594	14.936	18.396	18.232	15.730	18.033	21.969
338.15	20.117	23.815	27.005	31.369	34.113	22.850	15.105	18.605	18.439	15.908	18.238	22.218
343.15	20.384	24.131	27.363	31.786	34.566	23.154	15.306	18.852	18.684	16.119	18.480	22.513
348.15	19.264	22.805	25.860	30.039	32.666	21.881	14.465	17.816	17.658	15.234	17.465	21.276
353.15	18.174	21.514	24.396	28.339	30.817	20.643	13.646	16.808	16.658	14.371	16.476	20.072
363.15	16.682	19.748	22.394	26.013	28.288	18.949	12.526	15.428	15.291	13.192	15.124	18.424

373.15	15.306	18.119	20.547	23.868	25.955	17.386	11.493	14.156	14.030	12.104	13.877	16.905
378.15	15.017	17.777	20.158	23.416	25.464	17.057	11.275	13.888	13.764	11.875	13.614	16.585
383.15	14.868	17.601	19.959	23.185	25.212	16.889	11.164	13.751	13.628	11.758	13.480	16.421
388.15	15.275	18.083	20.505	23.819	25.902	17.351	11.470	14.127	14.001	12.079	13.848	16.870
393.15	16.380	19.390	21.988	25.541	27.775	18.605	12.299	15.149	15.014	12.953	14.850	18.090
398.15	17.550	20.775	23.558	27.365	29.759	19.934	13.177	16.230	16.086	13.878	15.910	19.382
403.15	16.428	19.447	22.052	25.616	27.856	18.660	12.335	15.193	15.058	12.991	14.893	18.143
408.15	15.118	17.897	20.294	23.574	25.636	17.172	11.352	13.982	13.857	11.955	13.706	16.697
413.15	13.956	16.521	18.734	21.761	23.664	15.852	10.479	12.907	12.792	11.036	12.652	15.413
423.15	13.182	15.605	17.695	20.555	22.353	14.973	9.898	12.191	12.083	10.424	11.951	14.559
433.15	13.463	15.937	18.072	20.993	22.828	15.292	10.109	12.451	12.340	10.646	12.205	14.868
443.15	13.581	16.077	18.231	21.177	23.029	15.426	10.197	12.560	12.448	10.739	12.312	14.999
453.15	12.096	14.319	16.237	18.861	20.510	13.739	9.082	11.186	11.087	9.565	10.966	13.359
463.15	11.610	13.744	15.586	18.105	19.688	13.188	8.718	10.738	10.642	9.181	10.526	12.823
473.15	10.164	12.032	13.644	15.849	17.235	11.545	7.632	9.400	9.316	8.037	9.215	11.225

Table S16. Values (in kJ/mol) of the London free dispersive interaction energy ($-\Delta G_a^d(T)$ of organic solvents adsorbed on PMMA/silica for $\theta = 0.83$ at different temperatures.

T(K)	C5	C6	C7	C8	C9	CCl4	CH2Cl2	CHCl3	Diethyl ether	THF	Ethyl acetate	Toluene
303.15	19.989	23.662	26.833	31.169	33.895	22.705	15.009	18.486	18.322	15.807	18.122	22.076
313.15	19.173	22.697	25.737	29.897	32.511	21.778	14.396	17.732	17.574	15.161	17.382	21.175
323.15	18.025	21.338	24.196	28.107	30.565	20.474	13.534	16.670	16.522	14.254	16.341	19.907
328.15	18.434	21.822	24.746	28.745	31.259	20.939	13.841	17.049	16.897	14.577	16.712	20.359
333.15	19.158	22.679	25.718	29.874	32.486	21.761	14.385	17.718	17.560	15.150	17.369	21.159
338.15	18.596	22.014	24.963	28.997	31.533	21.122	13.963	17.198	17.045	14.705	16.859	20.538
343.15	17.912	21.204	24.045	27.931	30.374	20.346	13.450	16.566	16.418	14.165	16.239	19.783
348.15	17.162	20.317	23.038	26.762	29.102	19.494	12.887	15.872	15.731	13.572	15.559	18.955
353.15	16.295	19.289	21.874	25.409	27.631	18.508	12.235	15.070	14.936	12.885	14.773	17.996
363.15	15.034	17.797	20.181	23.442	25.492	17.076	11.288	13.904	13.780	11.888	13.629	16.604
373.15	13.379	15.837	17.959	20.862	22.686	15.196	10.045	12.373	12.263	10.579	12.129	14.776
378.15	12.926	15.302	17.352	20.156	21.919	14.682	9.706	11.955	11.848	10.222	11.719	14.276
383.15	14.022	16.599	18.822	21.864	23.776	15.927	10.528	12.968	12.852	11.088	12.712	15.486
388.15	17.032	20.162	22.863	26.558	28.880	19.346	12.788	15.752	15.611	13.468	15.441	18.810
393.15	15.482	18.328	20.783	24.142	26.253	17.586	11.625	14.318	14.191	12.243	14.036	17.099
398.15	14.457	17.114	19.406	22.543	24.514	16.421	10.855	13.370	13.251	11.432	13.106	15.966
403.15	13.654	16.163	18.328	21.291	23.152	15.509	10.252	12.627	12.515	10.797	12.378	15.079
408.15	12.510	14.810	16.794	19.508	21.214	14.210	9.393	11.570	11.467	9.893	11.342	13.817
413.15	11.428	13.528	15.341	17.820	19.378	12.981	8.581	10.569	10.475	9.037	10.360	12.621
423.15	11.099	13.139	14.899	17.307	18.820	12.607	8.334	10.265	10.173	8.777	10.062	12.258
433.15	13.050	15.448	17.517	20.349	22.128	14.823	9.798	12.069	11.961	10.319	11.831	14.412
443.15	10.863	12.860	14.583	16.939	18.421	12.339	8.157	10.047	9.957	8.590	9.849	11.998

453.15	9.888	11.705	13.273	15.419	16.767	11.231	7.424	9.145	9.063	7.819	8.964	10.920
463.15	9.516	11.265	12.774	14.839	16.136	10.809	7.145	8.801	8.722	7.525	8.627	10.510
473.15	8.272	9.793	11.105	12.899	14.027	9.396	6.211	7.651	7.582	6.542	7.500	9.136

Table S17. Values (in kJ/mol) of the London free dispersive interaction energy ($-\Delta G_a^d(T)$ of organic solvents adsorbed on PMMA/silica for $\theta = 1.0$ (monolayer) at different temperatures.

T(K)	C5	C6	C7	C8	C9	CCl4	CH2Cl2	CHCl3	Diethyl ether	THF	Ethyl acetate	Toluene
303.15	19.008	22.501	25.516	29.639	32.231	21.590	14.272	17.579	17.423	15.031	17.232	20.993
313.15	18.022	21.335	24.193	28.103	30.560	20.471	13.532	16.668	16.519	14.252	16.339	19.904
323.15	16.901	20.007	22.687	26.354	28.659	19.197	12.690	15.631	15.491	13.365	15.322	18.666
328.15	17.360	20.550	23.303	27.069	29.437	19.718	13.035	16.055	15.912	13.728	15.738	19.172
333.15	18.192	21.536	24.421	28.368	30.849	20.664	13.660	16.825	16.675	14.386	16.493	20.092
338.15	17.475	20.686	23.457	27.249	29.631	19.849	13.121	16.161	16.017	13.818	15.842	19.299
343.15	19.336	22.889	25.956	30.151	32.787	21.963	14.518	17.882	17.723	15.290	17.530	21.355
348.15	19.617	23.223	26.334	30.590	33.265	22.283	14.730	18.143	17.981	15.513	17.785	21.666
353.15	15.341	18.160	20.593	23.921	26.013	17.425	11.519	14.188	14.061	12.131	13.908	16.943
363.15	14.091	16.681	18.916	21.973	23.895	16.006	10.581	13.032	12.916	11.143	12.775	15.563
373.15	12.389	14.666	16.631	19.319	21.008	14.072	9.303	11.458	11.356	9.797	11.232	13.683
378.15	12.160	14.395	16.324	18.962	20.620	13.813	9.131	11.246	11.146	9.616	11.024	13.430
383.15	15.057	17.825	20.213	23.479	25.532	17.103	11.306	13.925	13.801	11.907	13.651	16.630
388.15	17.892	21.180	24.017	27.899	30.339	20.322	13.434	16.547	16.399	14.148	16.220	19.760
393.15	16.704	19.774	22.423	26.047	28.324	18.973	12.542	15.448	15.311	13.209	15.143	18.448
398.15	15.382	18.209	20.649	23.986	26.084	17.472	11.550	14.226	14.099	12.164	13.945	16.989
403.15	13.446	15.917	18.049	20.966	22.800	15.272	10.096	12.435	12.324	10.633	12.190	14.850
408.15	12.147	14.379	16.306	18.941	20.597	13.797	9.121	11.234	11.134	9.605	11.012	13.415
413.15	11.159	13.209	14.979	17.400	18.921	12.675	8.379	10.320	10.228	8.824	10.116	12.324
423.15	10.935	12.945	14.679	17.051	18.542	12.420	8.211	10.113	10.023	8.647	9.913	12.077
433.15	12.745	15.088	17.109	19.874	21.612	14.477	9.570	11.787	11.682	10.079	11.555	14.076
443.15	10.190	12.063	13.679	15.890	17.280	11.575	7.652	9.425	9.341	8.058	9.239	11.255
453.15	8.844	10.469	11.872	13.790	14.996	10.045	6.640	8.179	8.106	6.993	8.018	9.767
463.15	8.491	10.052	11.398	13.240	14.398	9.645	6.376	7.853	7.783	6.714	7.698	9.378
473.15	7.424	8.788	9.966	11.576	12.589	8.432	5.574	6.866	6.805	5.871	6.730	8.199

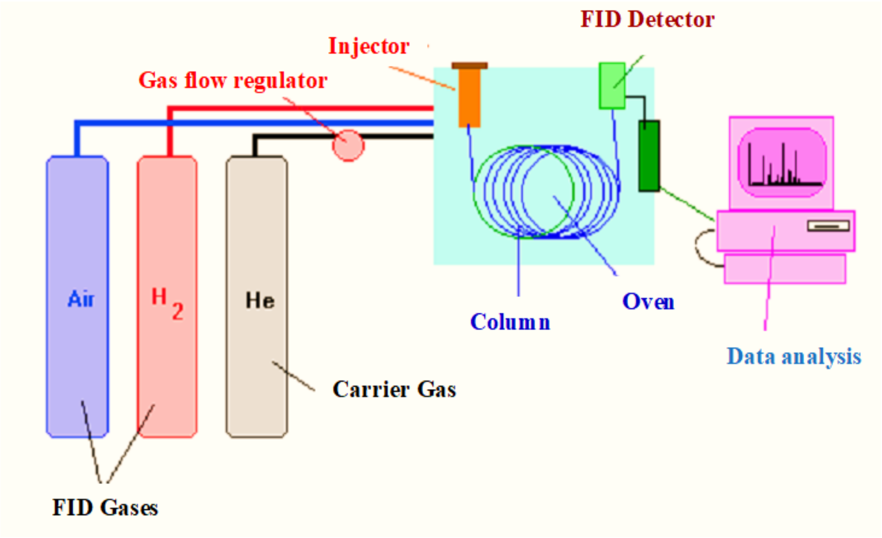

Figure S16. Elements of a gas chromatograph
